# New Phenolic Dimers from Plant *Paeonia suffruticosa* and Their Cytotoxicity and NO Production Inhibition

**DOI:** 10.3390/molecules28124590

**Published:** 2023-06-06

**Authors:** Qianqian Meng, Shunyao Tong, Yuqing Zhao, Xingrong Peng, Zhenghui Li, Tao Feng, Jikai Liu

**Affiliations:** 1 School of Pharmaceutical Sciences, South-Central Minzu University, Wuhan 430074, China; 2020201102012@stu.ahtcm.edu.cn (Q.M.); 2021120647@mail.scuec.edu.cn (S.T.); zyq@stu.ahtcm.edu.cn (Y.Z.);; 2 School of Pharmacy, Anhui University of Chinese Medicine, Hefei 230012, China; 3 Medical School, Fuyang Normal University, Fuyang 236037, China; 4 State Key Laboratory of Phytochemistry and Plant Resources in West China, Kunming Institute of Botany, Chinese Academy of Sciences, Kunming 650201, China; pengxingrong@mail.kib.ac.cn

**Keywords:** *Paeonia suffruticosa*, benzofuranones, cytotoxicity, NO production inhibition

## Abstract

The *Paeonia suffruticosa*, known as ‘Feng Dan’, has been used for thousands of years in traditional Chinese medicine. In our chemical investigation on the root bark of the plant, five new phenolic dimers, namely, paeobenzofuranones A–E (**1**–**5**), were characterized. Their structures were determined using spectroscopic analysis including 1D and 2D NMR, HRESIMS, UV, and IR, as well as ECD calculations. Compounds **2**, **4**, and **5** showed cytotoxicity against three human cancer cell lines, with IC_50_ values ranging from 6.7 to 25.1 μM. Compounds **1** and **2** showed certain inhibitory activity on NO production. To the best of our knowledge, the benzofuranone dimers and their cytotoxicity of *P. suffruticosa* are reported for the first time in this paper.

## 1. Introduction

*Paeonia suffruticosa* is a perennial deciduous shrub belonging to the family Paeoniaceae. The dried root bark of *P. suffruticosa* is called ‘Feng Dan’ or ‘Mudanpi’ in China, which is used as a traditional Chinese medicine to clear pathogenic heat from the blood and promote blood circulation to remove blood stasis, as recorded in the *Chinese Pharmacopoeia* (2020 edition). Currently, pharmacological studies on *P. suffruticosa* demonstrate anti-inflammatory, antioxidant, and anti-tumor activities, as well as central nervous system and cardiovascular system protective activities [1,2,3]. In order to understand the bioactive metabolites, *P. suffruticosa* was chemically investigated, and more than 190 constituents were reported in the past ten years including phenolics, monoterpenes and glycosides, flavonoids, triterpenes, sesquiterpenes, and lignans. A pharmaceutical investigation on these metabolites demonstrated promising anti-inflammatory and anti-tumor properties; the representatives are paeonisides A and B, mudanpiosides C and F, and suffruticosol A [4,5,6,7,8]. 

**Figure 1:**
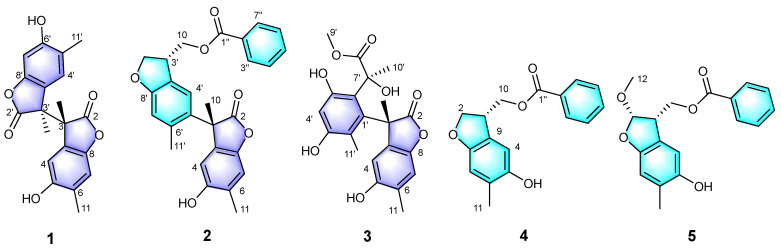
Structures of compounds **1**–**5**.

In order to further clarify the chemical constituents and biological activities of genuine medicinal materials of ‘Feng Dan’ from Tongling (China), as well as part of our ongoing work on bioactive natural products from natural sources [9,10,11,12,13,14], the study on the chemical constituents of the root bark of *P. suffruticosa* was carried out. As a result, five new phenolics were isolated, namely, paeobenzofuranones A–E (**1**–**5**). Their structures (Figure 1) were determined using extensive spectroscopic methods. All compounds were evaluated for their cytotoxicity against three human cancer cell lines including breast cancer MDA-MB-231, human myeloid leukemia HL-60, and colon cancer SW480. In addition, their anti-inflammatory activity by inhibiting NO production was also evaluated. Herein, the isolation, structural elucidation, and bioactivities of the compounds from *P. suffruticosa* are reported.

## 2. Results and Discussion

### 2.1. Structural Elucidation of Compounds ***1**–**5***

**Figure 2:**
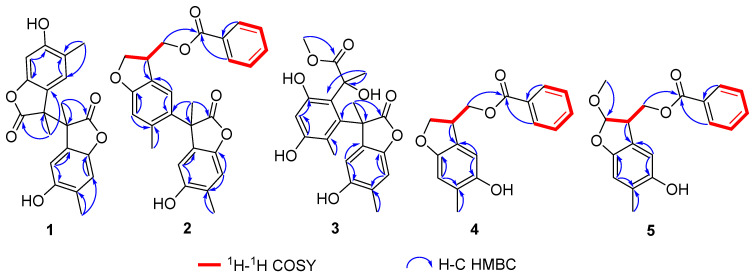
Selected HMBC and ^1^H–^1^H COSY correlations of compounds **1**–**5**.

**Figure 3:**
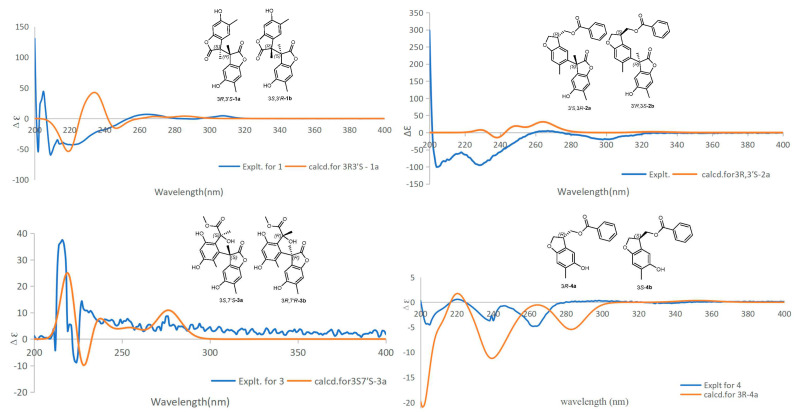
ECD calculations for compounds **1**–**4**.

Compound **1** was obtained as a colorless oily liquid. Its molecular formula was determined as C_20_H_18_O_6_ by the HR-ESI-MS with a molecular ion peak at *m*/*z* 377.09952 ([M + Na]^+^, calcd. 377.10011). The UV absorption peaks at λ_max_ 290 and 230 nm indicated the presence of a conjugated system. The IR spectrum indicated that compound **1** possessed hydroxyl (3406 cm^−1^) and lactone (1716 cm^−1^) groups. The ^1^H NMR spectroscopic data (Table 1) of compound **1** showed two methyl signals at *δ*_H_ 1.67 (s, 3H) and 2.15 (s, 3H), and two singlets for aromatic protons at *δ*_H_ 6.61 (1H, s, H-7) and 7.18 (1H, s, H-4). The interpretation of the ^13^C NMR (Table 2) and DEPT spectra data displayed 10 carbon signals, which indicated six non-protonated carbons (C-2: *δ*_C_ 179.4, C-3: *δ*_C_ 52.6, C-5: *δ*_C_ 113.3, C-6: *δ*_C_ 153.7, C-8: *δ*_C_ 146.2, C-9: *δ*_C_ 127.4), two CH (C-4: *δ*_C_ 128.1, C-7: *δ*_C_ 110.3), and two CH_3_ (C-10: *δ*_C_ 18.7, C-11: *δ*_C_ 16.6). A preliminary analysis of these data suggested that compound **1** should be a benzofuranone derivative with a structure similar to that of 4,6-dihydroxy-3,5-dimethylcoumaran-2-one [15]. The presence of a methyl at *δ*_C_ 18.7 and a carbonyl carbon at *δ*_C_ 179.4 indicated the differences. In the HMBC spectrum (Figure 2), correlations from H_3_-11 (*δ*_H_ 2.11) to C-4 (*δ*_C_ 128.1), C-5 (*δ*_C_ 113.3), and C-6 (*δ*_C_ 153.7), and from H_3_-10 (*δ*_H_ 1.67) to C-3 (*δ*_C_ 52.6), C-2 (*δ*_C_ 179.4), and C-9 (*δ*_C_ 127.4) enabled the assignment of the methyl and the carbonyl carbon. A further analysis of the ^13^C NMR data for a quaternary carbon at C-3 (*δ*_C_ 52.6) revealed that compound **1** should be a symmetric dimer with a linkage by the bond of C-3/C-3′. It was also supported by the MS data analysis. The absolute configurations of C-3 and C-3′ were identified as 3*R* and 3′*S* by the ECD calculations (Figure 3). Finally, the structure of compound **1** was identified and trivially named as paeobenzofuranone A.

Compound **2** was obtained as a white powder. Its molecular formula was determined as C_27_H_24_O_6_ by HR-ESI-MS (measured at *m*/*z* 445.16489 [M + Na]^+^; calcd. 445.16511). The UV spectrum revealed the conjugated system by peaks at λ_max_ 295 and 230 nm. The 1D spectra data of compound **2** (Table 1 and Table 2) are partially identical to those of compound **1**. The interpretation of the ^1^H and ^13^C spectroscopic data of compound **2** showed two benzofuran parts and an additional benzoyl moiety. The locations of the benzofuran parts were assigned by the HMBC correlations from H-10 (*δ*_H_ 1.75) to C-3 (*δ*_C_ 52.6), C-6 (*δ*_C_ 126.3), and C-2 (*δ*_C_ 179.3), as well as from H-4 (*δ*_H_ 6.61) to C-5 (*δ*_C_ 126.3) and C-3 (*δ*_C_ 52.6) (Figure 2). Furthermore, the location of the benzoyl was assigned by the key HMBC peaks from H-3′ (*δ*_H_ 3.81) to C-10′ (*δ*_C_ 66.9); from H-2′ (*δ*_H_ 4.43) to C-3′ (*δ*_C_ 43.5) and C-2′ (*δ*_C_ 73.1); and from H-10′ (*δ*_H_ 4.44) to C-3′ (*δ*_C_ 43.5) and C-10′ (*δ*_C_ 66.9). The absolute configurations of C-3 and C-3′ were established as 3*S* and 3′*R* by the ECD calculations (Figure 3). Then, the structure of compound **2** was established and named as paeobenzofuranone B.

Compound **3** was obtained as a white powder. The IR spectrum indicated that compound **3** possessed hydroxyl (3394 cm^−1^) and lactone (1712 cm^−1^) groups. Its molecular formula was determined as C_21_H_22_O_8_ by the HR-ESI-MS data analysis (*m*/*z* 425.12030 ([M + Na]^+^, calcd. 425.12124). The ^1^H and ^13^C NMR spectra data of compound **3** (Table 1 and Table 2) are partially the same as those of compound **1**. The interpretation of the ^1^H and ^13^C spectroscopic data of compound **3** revealed one benzofuran part and one benzene ring. The benzofuran part was assigned by the HMBC correlations from H-10 (*δ*_H_ 1.75) to C-3 (*δ*_C_ 50.2), C-1′ (*δ*_C_ 133.7), and C-9 (*δ*_C_ 126.4); from H-11 (*δ*_H_ 2.02) to C-7 (*δ*_C_ 118.9), C-9 (*δ*_C_ 126.4), and C-5 (*δ*_C_ 149.2); and from H-4′ (*δ*_H_ 6.88) to C-2′ (*δ*_C_ 153.2) and C-5′ (*δ*_C_ 144.6) (Figure 2). The ^1^H-^1^H COSY cross peaks from *δ*_H_ 2.02 (3H, s, H-11) to *δ*_H_ 6.33 (1H, s, H-7), and from *δ*_H_ 1.77 (3H, s, H-11′) to *δ*_H_ 6.88 (1H, s, H-4′) verified the location of 10-CH_3_ and 11-CH_3_. The HMBC correlations verified the benzofuran group attached to C-8 (*δ*_C_ 144.3). Furthermore, from the HMBC correlations, the signal of another ester carbonyl group was connected to the benzene ring through the C-7′ (*δ*_C_ 75.1), as evidenced from *δ*_H_ 1.63 (3H, s, H-10′) to *δ*_C_ 75.1 (CH, C-7′), and from *δ*_H_ 3.73 (OCH_3_, s, H-9′) to *δ*_C_ 176.8 (C, C-8′). The absolute configurations of C-3 and C-7′ were established as 3*S* and 7′*S* by the ECD calculations (Figure 3). Eventually, the structure of compound **3** was elucidated as paeobenzofuranone C.

**Figure 4:**
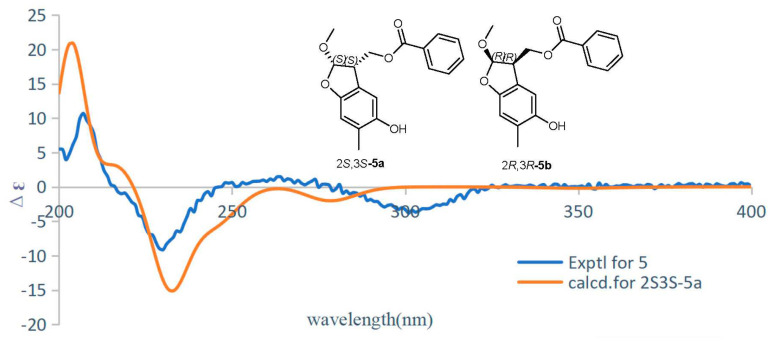
ECD calculations for compound **5**.

Compound **4** was obtained as a white powder. The IR spectrum indicated that compound **4** possessed hydroxyl (3383 cm^−1^) and lactone (1708 cm^−1^) groups. Its molecular formula was determined as C17H16O4 by the HR-ESI-MS data analysis (*m*/*z* 285.11215 ([M + H]^+^, calcd. 285.11268). The ^1^H and ^13^C spectra data of compound **4** (Table 1 and Table 2, Supplementary data) are partially the same as those of compound **1**, except for the benzoyl and hydroxymethyl groups in compound **4**. The interpretation of the ^1^H and ^13^C spectroscopic data of compound **4** implied one benzofuran part and one benzoyl. The locations of the benzofuran parts were assigned by the correlations revealed in the HMBC experiment (Figure 2) between the 11-CH_3_ (*δ*_H_ 2.14) and C-5 (*δ*_C_ 148.9), C-7 (*δ*_C_ 110.6), and C-8 (*δ*_C_ 153.4); as well as from H-4 (*δ*_H_ 6.74) to C-5, C-8, and C-9 (*δ*_C_ 125.6); from H-3 (*δ*_H_ 3.81) to C-10 (*δ*_C_ 66.4); and from H-7 (*δ*_H_ 4.37) to C-1′′ (*δ*_C_ 166.5). The ^1^H-^1^H COSY correlations from *δ*H 3.81 (1H, s, H-3) to *δ*H 4.43 (1H, s, H-10), and from *δ*H 4.43 (1H, s, H-10) to *δ*H 6.74 (1H, s, H-4) verified the location of the benzofuran part and one benzoyl connecting by C-3 and C-10. The absolute configuration of C-3 was established as 3*R* by the ECD calculations (Figure 4). Therefore, the structure of compound **4** was elucidated as paeobenzofuranone D.

Compound **5** was obtained as a white powder. Its molecular formula was determined as C_18_H_18_O_5_ by the HR-ESI-MS data analysis (*m*/*z* 313.10959 [M − H]^−^, calcd. 313.10743). The ^1^H and ^13^C NMR data resembled those of compound **4** (Table 1 and Table 2), except for the presence of an additional methoxy at C-2 in compound **5**, which was confirmed by the key HMBC correlation of H-12 (*δ*H 3.48) with C-2 (*δ*_C_ 111.2). A comprehensive analysis of the 2D NMR data indicated that other parts of compound **5** were the same as those of compound **4**. The absolute configurations of C-2 and C-3 were established as 2*S* and 3*S* by the ECD calculations (Figure 4). Thus, the structure of compound **5** was established as paeobenzofuranone E.

### 2.2. Bioactivity Analysis

Five new compounds were tested for their inhibitory activities on nitric oxide production in the model of lipopolysaccharide-activated macrophages. As shown in Table 3, compounds **1** and **2** showed comparable inhibitory activity with the positive control at the concentration of 50 μM. In addition, all compounds were evaluated for their cytotoxicity against the HL-60, SW480, and MDA-MB-231 cell lines. As shown in Table 4, compounds **2**, **4**, and **5** demonstrated cytotoxicity against three human cancer cell lines. In particular, they exhibited potent cytotoxicity against HL-60 cells, with IC_50_ values of 6.8, 19.1, and 11.1 μM, compared to those of the positive control. In addition, compounds **4** and **5** showed no cytotoxicity to MDA-MB-231, indicating selectivity to the cancer cell lines. 

## 3. Experiments

### 3.1. General Experimental Procedures

The UV spectra were obtained on a UH5300 UV-VIS Double Beam Spectrophotometer. The IR spectra were accessed using a Shimadzu Fourier transform infrared spectrometer with KBr pellets. The HRESIMS were measured on a Thermo Scientific Q Exactive Orbitrap mass spectrometer system. The NMR spectra (^1^H, ^13^C, and 2D NMR) were run on a Bruker Avance III NMR instrument at 600 MHz for ^1^H and 150 MHz for ^13^C NMR, while tetramethylsilane (TMS) was used as an internal standard. Column chromatography (CC) was executed on silica gel (200−300 mesh, Qingdao Marine Chemical Ltd., Qingdao, China), Sephadex LH-20 (Pharmacia Fine Chemical Co., Ltd., Stockholm, Sweden), and reverse phase silica gel (20−45 μm, Fuji Silysia Chemical Ltd., Kasugai, Japan). Medium pressure liquid chromatography (MPLC) was applied to Biotage SP2 equipment, and the columns were packed with reverse phase silica gel (C_18_). An Agilent 1260 series high-performance liquid chromatography (HPLC) system was used for the sample analysis (ZORBAX-SB C_18_ column, 5 μm, 4.6 × 250 mm, flowing rate = 1 mL/min) and preparation (ZORBAX-SB C_18_ column, 5 μm, 9.4 × 150 mm, flowing rate = 6 mL/min). All fractions were monitored using thin-layer chromatography (TLC) over GF 254 and silica gel 60 plates. The spots were visualized by using heating silica gel plates soaked with vanillin–sulfuric acid color component solvent.

### 3.2. Plant Material

The root barks of *P. suffruticosa* were collected in August 2021 from Tongling County, Anhui Province, People’s Republic of China. It was identified by Zhenghui Li. (Associate Professor of South-Central Minzu University, Wuhan, China). A voucher specimen (2021123 FD) was deposited at the School of Pharmaceutical Sciences, South-Central Minzu University.

### 3.3. Extraction and Isolation

The root bark of *P. suffruticosa* (50 kg) were mechanically crushed and extracted with MeOH/H_2_O (80:20) at 52 °C four times. The solvent was evaporated in vacuo to obtain a dark gum (9.3 kg). The latter was dissolved in a liter of water and then, respectively, extracted with petroleum ether (PE, 8L × 4) and dichloromethane (DCM, 8L × 4) to obtain PE (1.3 kg) and DCM parts (560 g). The DCM part was separated using a silica gel column eluted with PE: acetone (50:1, 40:1, 30:1, 20:1, 10:1) to obtain eight fractions (TPG-1–8). The fraction TPG-3 (28.5 g) was subjected to ODS silica gel CC and eluted with MeOH/H_2_O (20:90→90:10, *v*/*v*) to yield 10 fractions (Fr. 3-1→3-10). Fr. 3-2 (210 mg) was purified using Sephadex LH-20 (MeOH:DCM = 1:1) to obtain three fractions (Fr. 3-2-1, 3-2-2, 3-2-1). Fr. 3-2-3 was purified using preparative HPLC with CH_3_CN/H_2_O (30:70→60:40, *v*/*v*, 30 min) to obtain compound **1** (15.6 mg, t*_R_* = 12.5 min), compound **2** (9.7 mg, t*_R_* = 14.8 min), compound **4** (3.3 mg, t*_R_* = 16.9 min), and compound **5** (4.9 mg, t*_R_* = 17.8 min), respectively. Fr. 3-2-1 was prepared using HPLC with CH_3_CN/H_2_O (37:63→60:40, *v*/*v*, 30 min) to obtain compound **3** (3.2 mg) at 17.8 min. 

#### 3.3.1. Paeobenzofuranone A (**1**)

Colorless oil; ${\left[\alpha \right]}_{D}^{26}$ −98.6 (*c* = 0.09, MeOH); UV (MeOH) *λ*_max_ (log ε): 230 (3.6) nm; IR (KBr) *ν*max 3406, 1716, 1450, 1346, 1315, 1276, 1026, 1049, and 713 cm^−1^; HRESIMS: *m*/*z* 377.09952 [M + Na]^+^, (calcd. for C20H18O6Na^+^, 377.10011). The ^1^H and ^13^C NMR data are displayed in Table 1 and Table 2.

#### 3.3.2. Paeobenzofuranone B (**2**)

White powder; UV (MeOH) *λ*_max_ (log ε): 230 (3.2) nm; ${\left[\alpha \right]}_{D}^{26}$ −15.1 (*c* = 0.09, MeOH); HRESIMS: *m*/*z* 445.16489 [M + H]^+^ (calcd. for C27H24O6^+^ 445.16511). ^1^H and ^13^C NMR data are displayed in Table 1 and Table 2.

#### 3.3.3. Paeobenzofuranone C (**3**)

White powder; ${\left[\alpha \right]}_{D}^{22}$ –5.6 (*c* = 0.12, MeOH); UV (MeOH) *λ*_max_ (log ε): 230 (3.2) nm; IR (KBr) *ν*_max_: 3394, 1712, 1450, 1346, 1315, 1276, 1176, and 1072 cm^−1^; HRESIMS: *m*/*z* 425.12030 ([M + Na]^+^ (calcd. for C21H22O8Na^+^, 425.12124). ^1^H and ^13^C NMR data are displayed in Table 1 and Table 2.

#### 3.3.4. Paeobenzofuranone D (**4**)

White powder; ${\left[\alpha \right]}_{D}^{22}$ –3.8 (*c* = 0.11, MeOH); UV (MeOH) *λ*_max_ (log ε): 230 (1.973) nm; IR (KBr) *ν*_max_: 3383, 1708, 1450, 1342, 1315, 1276, 1176, and 1072 cm^−1^; HRESIMS *m*/*z* 285.11215 ([M + H]^+^ (calcd. for C17H16O4^+^, 285.11268). ^1^H and ^13^C NMR data are displayed in Table 1 and Table 2.

#### 3.3.5. Paeobenzofuranone E (**5**)

White powder; ${\left[\alpha \right]}_{D}^{26}$ −13.8 (c = 0.09, MeOH); UV (MeOH) *λ*_max_ (log ε): 230 (3.136) nm; HRESIMS *m*/*z* 313.10959 ([M−H]^−^ (calcd. for C_18_H_18_O_5_^−^, 313.10743). ^1^H and ^13^C NMR data are displayed in Table 1 and Table 2.

### 3.4. Cytotoxicity Assay

The cytotoxicity for the isolates was evaluated using the MTS assay. Briefly, 1 × 10^5^ cells/mL from three human cancer cell lines, breast cancer MDA-MB-231, human myeloid leukemia HL-60, and colon cancer SW480, were seeded in 96-well plates. After 24 h incubation, the cells were treated with test compounds or cisplatin (DDP, positive control) at given concentrations (40, 8, 1.6, 0.32, 0.064 μM) for 48 h. The MTS was then added to each well, and the plates were stored for 4 h. The absorbance was read at 490 nm. The IC_50_ (50% concentration of inhibition) was calculated using the Reed–Muench method [16,17].

### 3.5. NO Inhibitory Activity Assays

The mouse mononuclear macrophages RAW264.7 were seeded into 96-well plates, induced, and stimulated with 1 μg/mL LPS; at the same time, five new compounds with different concentrations to be tested were added. The drug-free group and the L-NMMA positive drug group were set approximately equal as a comparison. After the cells were cultured overnight, the medium was taken to detect the production of NO, and the absorbance was measured at 570 nm. The MTS was added to the remaining medium for cell viability assays to exclude the toxic effects of the compounds on the cells. The assays were performed as triplicate batch experiments. The NO production inhibition rate (%) = (OD_570nm_ of non-drug treatment group−OD_570nm_ of sample group)/OD_570nm_ of non-drug treatment group × 100% [18,19].

### 3.6. ECD Calculations

The conformers of the five calculated compounds were generated via MMFF in ChemDraw. The ECD were calculated at the B3LYP/6-31+G(d,p) level in methanol with the PCM model. The calculated ECD curves and weighted ECD were all generated using SpecDis 1.71 based on the Boltzmann distribution theory, and the simulated spectra of all the predominant conformers were averaged to obtain the final conformationally averaged data [20]. All of the density functional theory (DFT) calculations were implemented using the Gaussian 16 software package with the Gaussian 09 default keyword. For the computational details of compounds **1**–**5**, see the Supplementary Materials.

## 4. Conclusions

In the present study, the chemical investigation on *Paeonia suffruticosa* results in the isolation of five new benzofuran compounds, containing rare dimers (compounds **1**–**3**) and hetero-dimers (compounds **4** and **5**). Their structures were determined using extensive spectroscopic methods. This work represents the first report of new benzofuran dimers of *P. suffruticosa* and their cytotoxicity and broadens the horizon of the structural diversity of *P. suffruticosa*.

## Figures and Tables

**Table 1:** ^1^H NMR spectroscopic data of compounds **1**–**5** in Methanol-*d*_4_ (600 MHz, *J* in Hz).

Position	1	2	3	4	5
2				4.46, m	5.48, t (1.8)
3				3.81, t (7.4)	3.80, t (7.5)
4	7.18, s	6.61, s	6.96, s	6.74, s	6.72, s
7	6.61, s	6.52, s	6.33, s	4.44, dd (10.7, 5.9) 4.37, dd (18.0, 9.1)	4.46, dd (11.1, 5.5) 4.33, dd (11.1, 7.8)
10	1.67, s, 3H	1.75, s, 3H	1.72, s, 3H	4.43, m	4.48, m
11	2.15, s, 3H	2.13, s, 3H	2.02, s, 3H	2.14, s, 3H	2.15, s, 3H
12					3.48, s
2′		4.43, m; 4.06, m		8.00, d (1.5)	7.97, d (1.5)
3′		3.81, t (7.5)		7.44, m	7.45, m
4′	7.18, s	6.63, s	6.88, s	7.58, m	7.61, m
5′	6.61, s	6.62, s		7.49, m	7.48, m
6′	1.67, s, 3H			8.01, d (1.3)	7.99, d (1.3)
7′	2.15, s, 3H	6.85, s			
9′			3.73, s		
10′		4.60, dd (18.0, 9.1) 4.44, dd (10.8, 5.9)	1.63, s, 3H		
11′		2.05, s, 3H	1.77, s, 3H		
3′′		8.01, d (1.2)			
4′′		7.48, t (7.8)			
5′′		7.56, m			
6′′		7.48, t (7.8)			
7′′		8.00, d (1.4)			

**Table 2:** ^13^C NMR spectroscopic data of compounds **1**–**5** in Methanol-*d*_4_ (150 MHz).

Position	1	2	3	4	5
2	179.4, C	179.3, C	182.7, C	73.5, CH_2_	111.2, CH
3	52.6, C	52.6, C	50.2, C	42.0, CH	50.5, CH
4	128.1, CH	112.2, CH	115.6, CH	110.8, CH	112.6, CH
5	113.3, C	149.3, C	149.2, C	148.9, C	150.9, C
6	153.7, C	126.3, C	125.0, C	128.5, CH	124.4, CH
7	110.3, CH	112.4, CH	118.9, CH	110.6, CH	112.3, CH
8	146.2, C	125.9, C	144.3, C	153.4, C	153.2, C
9	127.4, C	127.4, C	126.4, C	125.6, C	129.8, C
10	18.7, CH_3_	18.6, CH_3_	22.3, CH_3_	66.4, CH_2_	66.3, CH_2_
11	16.6, CH_3_	16.6, CH_3_	15.9, CH_3_	15.6, CH_3_	17.0, CH_3_
12					56.2, OCH_3_
1′			133.7, C		
2′	179.4, C	73.1, CH_2_	153.2, C		
3′	52.6, C	43.5, CH	122.0, C		
4′	128.1, CH	111.7, CH	115.9, CH		
5′	113.3, C	126.4, C	144.6, C		
6′	153.7, C	131.4, CH	125.7, C		
7′	110.3, CH	110.3, CH	75.1, C		
8′	146.2, C	154.9, C	176.8, C		
9′	127.4, C	124.4, C	53.2, OCH_3_		
10′	18.7, CH_3_	66.9, CH_2_	26.0, CH_3_		
11′	16.6, CH_3_	16.8, CH_3_	10.4, CH_3_		
1′′		168.1, C		166.5, C	167.9, C
2′′		130.7, C		129.7, C	131.2, C
3′′		129.7, CH		129.2, CH	130.4, CH
4′′		128.1, CH		128.2, CH	129.5, CH
5′′		134.5, CH		132.9, CH	130.7, CH
6′′		129.7, CH		128.2, CH	129.5, CH
7′′		128.1, CH		129.1, CH	130.4, CH

**Table 3:** Inhibitory activities of compounds **1**–**5** on NO production at 50 μM.

Compound	Inhibition Activity (100%)
L-NMMA ^a^	52.0 ± 1.96
**1**	43.9 ± 2.07
**2**	44.6 ± 0.52
**3**	13.0 ± 1.59
**4**	33.7 ± 2.24
**5**	30.9 ± 1.56

^a^ L-NMMA (NG-monomethyl-L-arginine, monoacetate salt) was used as the positive control.

**Table 4:** Cytotoxicity of compounds **2**, **4**, and **5** (IC_50_ ± SD, μM).

Compound	HL-60	MDA-MB-231	SW480
**2**	6.8 ± 0.11	20.9 ± 0.46	12.6 ± 0.73
**4**	19.1 ± 0.32	>40	8.9 ± 0.40
**5**	11.1 ± 1.61	>40	10.7 ± 0.43
DDP ^a^	23.5 ± 0.77	16.9 ± 1.19	25.1 ± 1.26

^a^ DDP (Cisplatin) was used as the positive control.

## Data Availability

All the data in this research are presented in the manuscript and Supplementary Materials.

## Supplementary Materials

The following supporting information can be downloaded at https://www.mdpi.com/article/10.3390/molecules28124590/s1, Figures S1–S35: HRESIMS, 1D & 2D NMR, ECD data of compounds **1**–**5**; Tables S1–S5: Energy analysis for conformers of **1**–**5** at B3LYP/6-31+G(d,p)level in the gas phase.
