# Supplementary material for: New Phenolic Dimers from Plant Paeonia suffruticosa and Their Cytotoxicity and NO Production Inhibition
Source: Molecules. 2023 Jun 6;28(12):4590. doi: 10.3390/molecules28124590 (PMC10303828; doi:10.3390/molecules28124590)
Supplement: Supplementary file 1 [file molecules-28-04590-s001.zip › Supporting information.pdf]

Supporting Information for

New phenolic dimers from plant *Paeonia suffruticosa* and their cytotoxicity and NO production inhibition

Qian-Qian Meng [1,2], Shun-Yao Tong [1], Yu-Qing Zhao [1], Xing-Rong Peng [3], Zheng-Hui Li [1], Tao Feng[1,*], Ji-Kai Liu[1,*]

[1] School of Pharmaceutical Sciences, South-Central Minzu University, Wuhan, Hubei 430074, People's Republic of China
[2] Medical school, Fuyang Normal University, Fuyang, 236037, People's Republic of China
[3] State Key Laboratory of Phytochemistry and Plant Resources in West China, Kunming Institute of Botany, Chinese Academy of Sciences, Kunming, 650201, People's Republic of China
* Correspondence: E-mails: tfeng@mail.scuec.edu.cn (T. Feng); liujikai@mail.scuec.edu.cn (J.K. Liu).

Corresponding author: tfeng@mail.scuec.edu.cn (T. Feng); liujikai@mail.scuec.edu.cn (J.K. Liu).

Contents of Supporting Information

- **Section S1. NMR and MS for compounds 1–5.**
  - **Figure S1-S4 NMR spectra of paeobenzofuranone A (1)**
  - **Figure S5 HRESIMS spectrum of paeobenzofuranone A (1)**
  - **Figure S6-S11 NMR spectra of paeobenzofuranone B (2)**
  - **Figure S12HRESIMS spectrum of paeobenzofuranone B (2)**
  - **Figure S13-S17 NMR spectra of paeobenzofuranone C (3)**
  - **Figure S18 HRESIMS spectra of paeobenzofuranone C (3)**
  - **Figure S16-S23 NMR spectra of paeobenzofuranone D (4)**
  - **Figure S24 HRESIMS spectra of paeobenzofuranone D (4)**
  - **Figure S25-S29 NMR spectra of paeobenzofuranone E (5)**
  - **Figure S30 HRESIMS spectra of paeobenzofuranone E (5)**
- **Section S2. Computational details for compounds 1–5.**
  - **Section S2-1. Computational details for paeobenzofuranone A (1) (ECD)**
  - **Section S2-2. Computational details for paeobenzofuranone B (2) (ECD)**
  - **Section S2-3. Computational details for paeobenzofuranone C (3) (ECD)**
  - **Section S2-4. Computational details for paeobenzofuranone D (4) (ECD)**
  - **Section S2-5. Computational details for paeobenzofuranone E (5) (ECD)**

Section S1. NMR and MS for compounds 1–5.

Figure S1. ^{1}H NMR (600 MHz, Methanol-d_4) spectrum of paeobenzofuranone A (1).

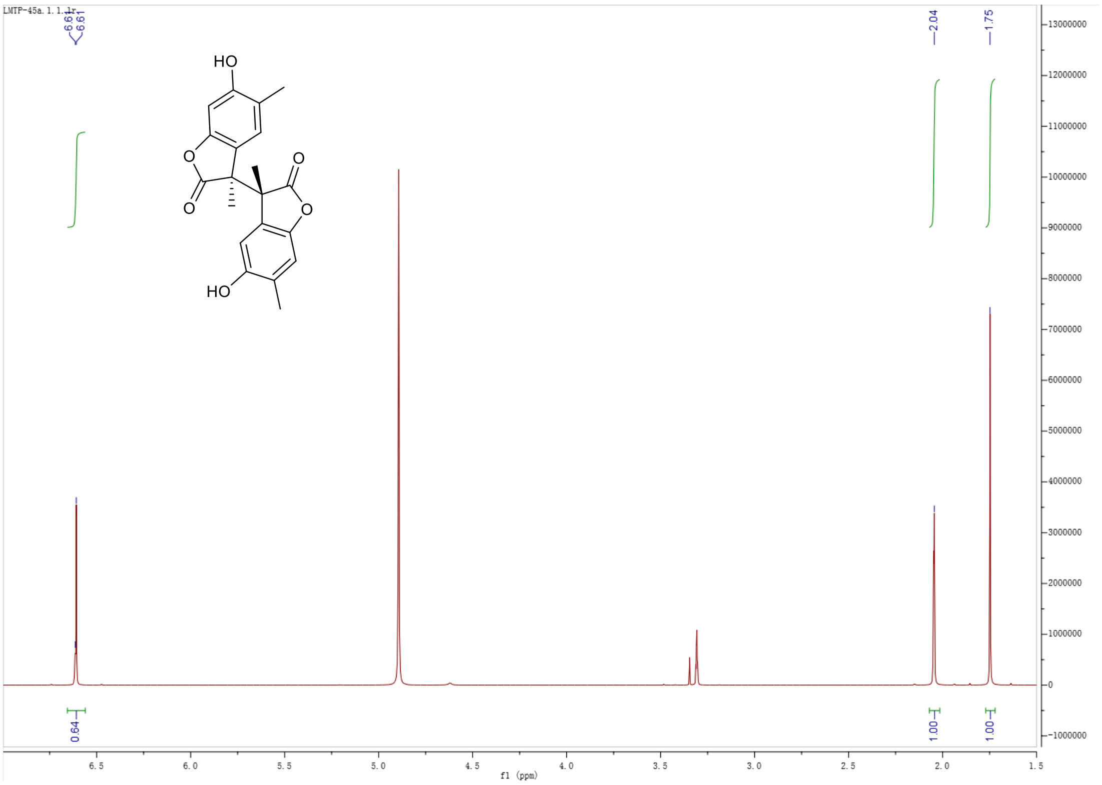

Figure S2. ^{13}C NMR (150 MHz, Methanol-d_4) spectrum of paeobenzofuranone A (1).

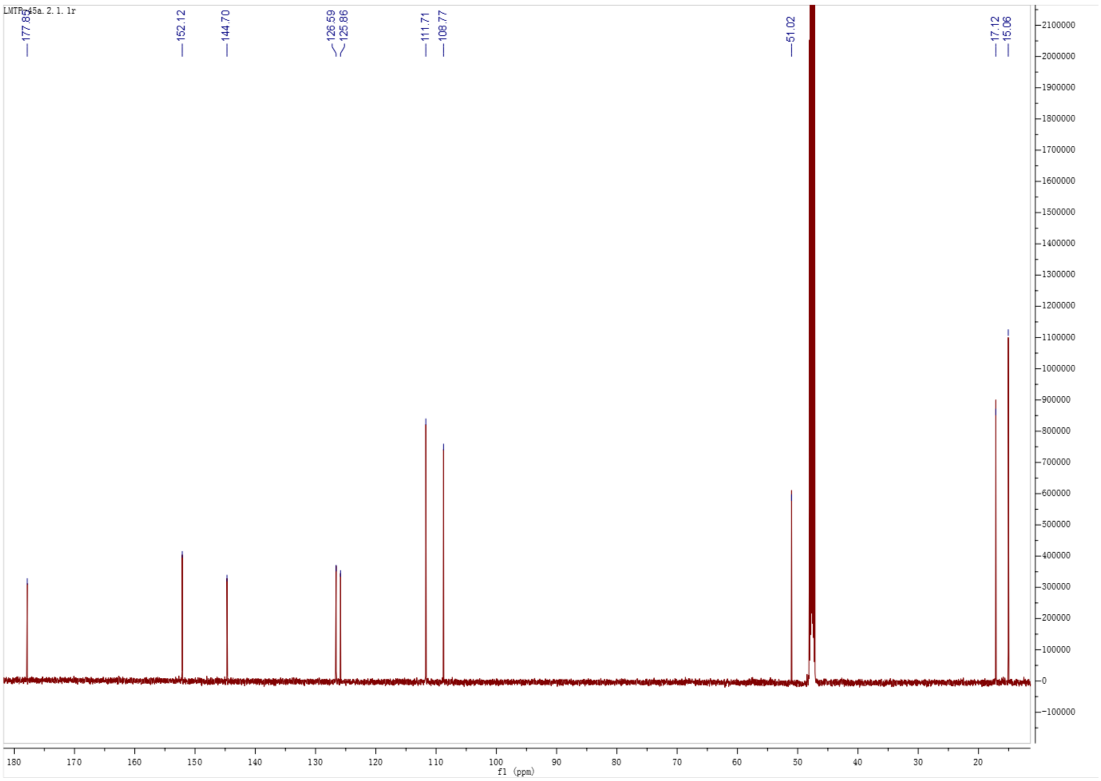

Figure S3. HSQC (600/150 MHz, Methanol-d_4) spectrum of paeobenzofuranone A (1).

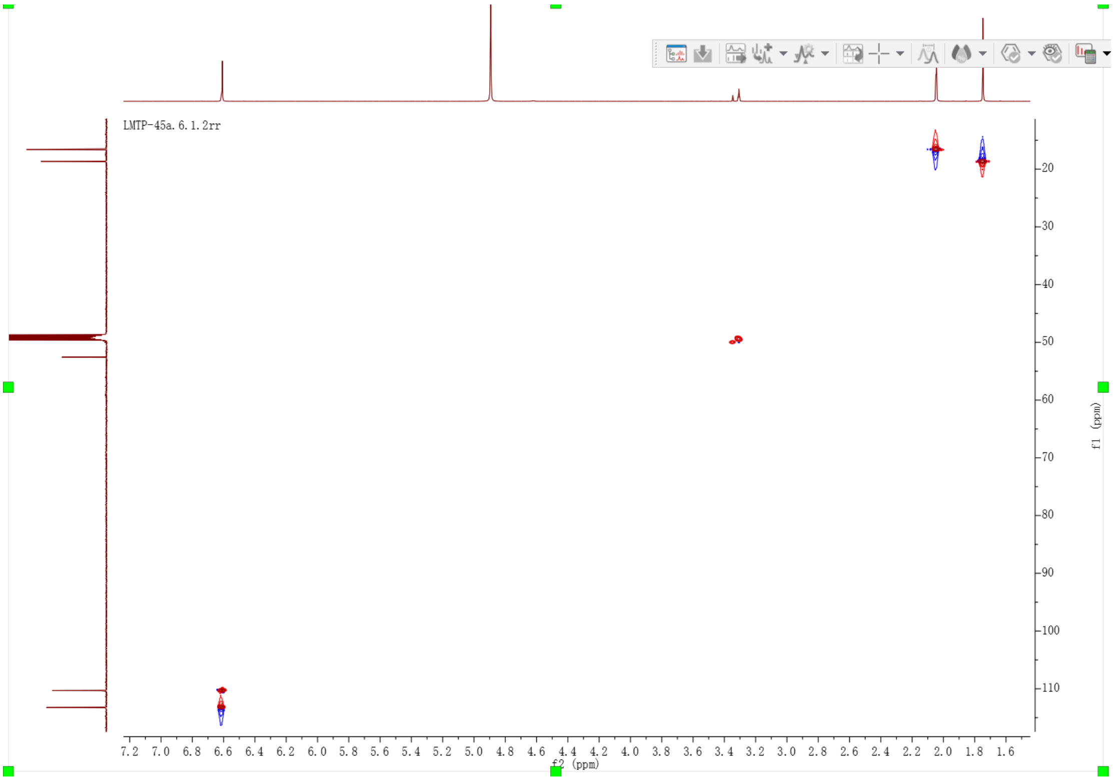

Figure S4. HMBC (600/150 MHz, Methanol-d_4) spectrum of paeobenzofuranone A (1).

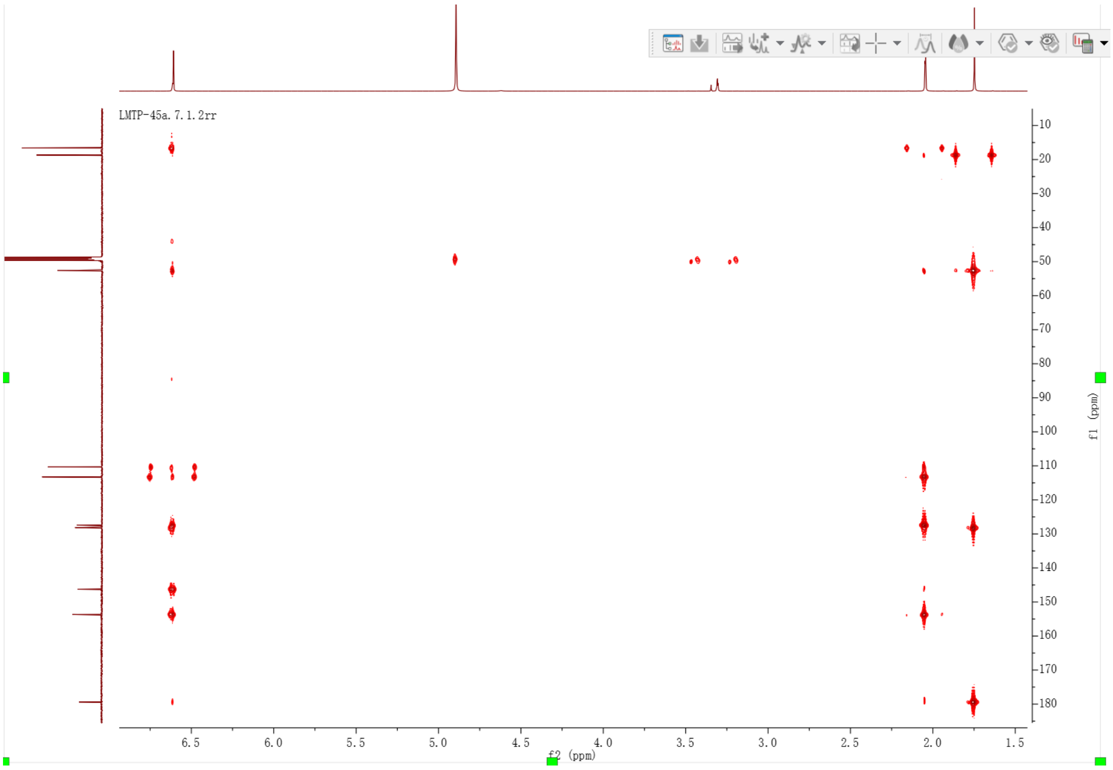

Figure S5. HRESIMS spectrum of paeobenzofuranone A (1)

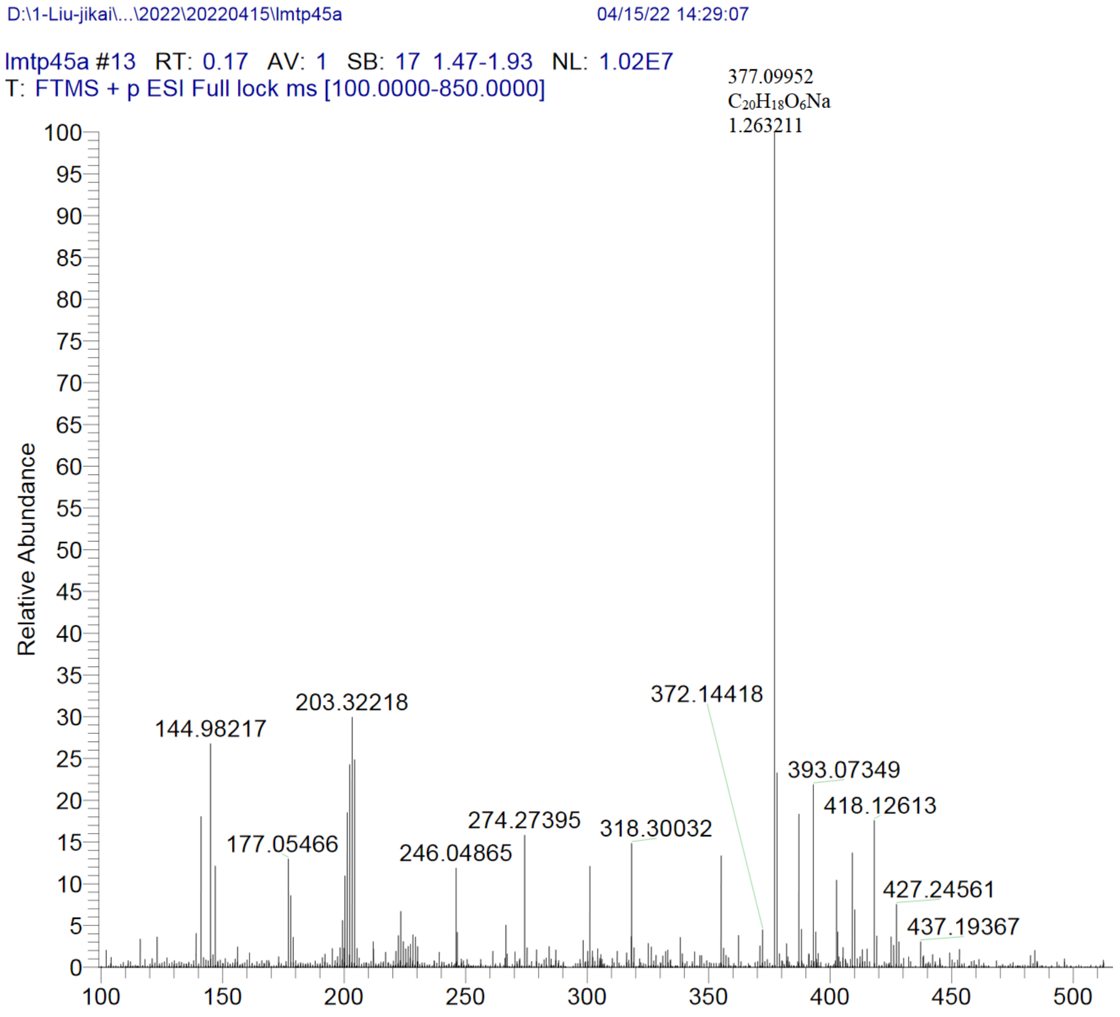

Figure S6. ^{1}H NMR (600 MHz, Methanol-d_4) spectrum of paeobenzofuranone B (2).

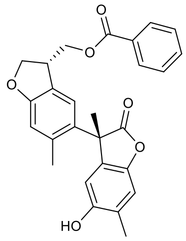

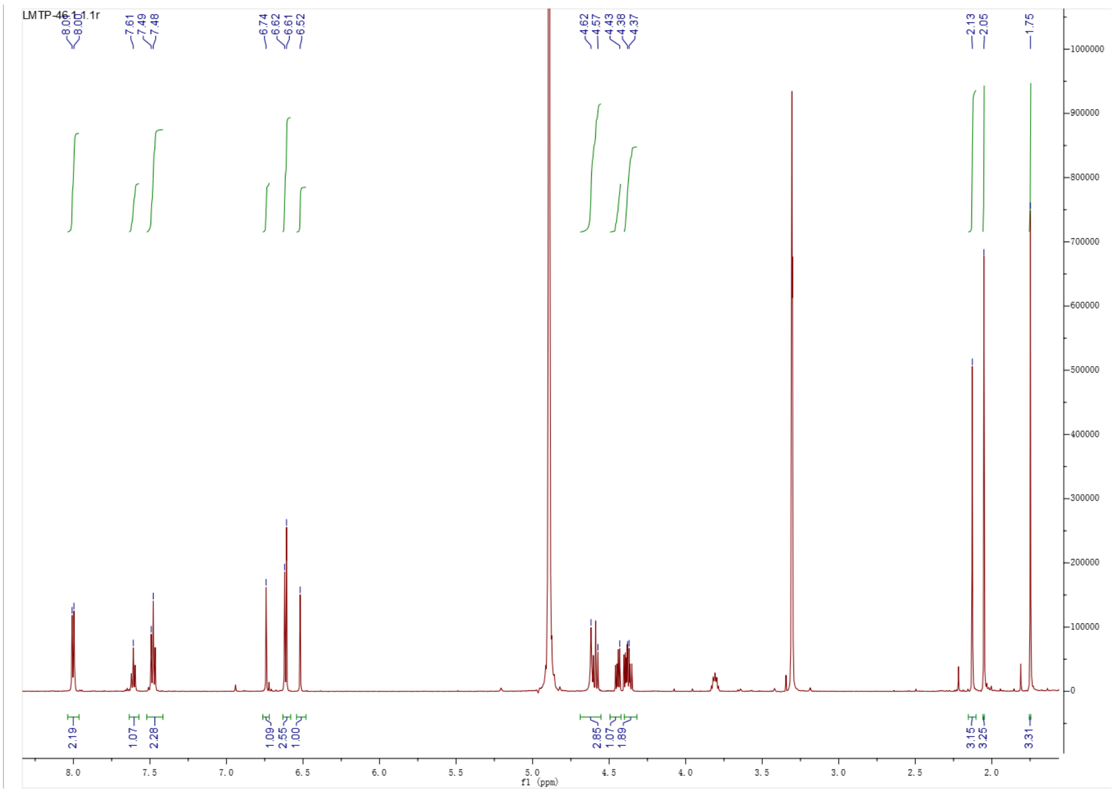

Figure S7. ^{13}**C NMR (150 MHz, Methanol-*d*$_4$) spectrum of paeobenzofuranone B (2).**

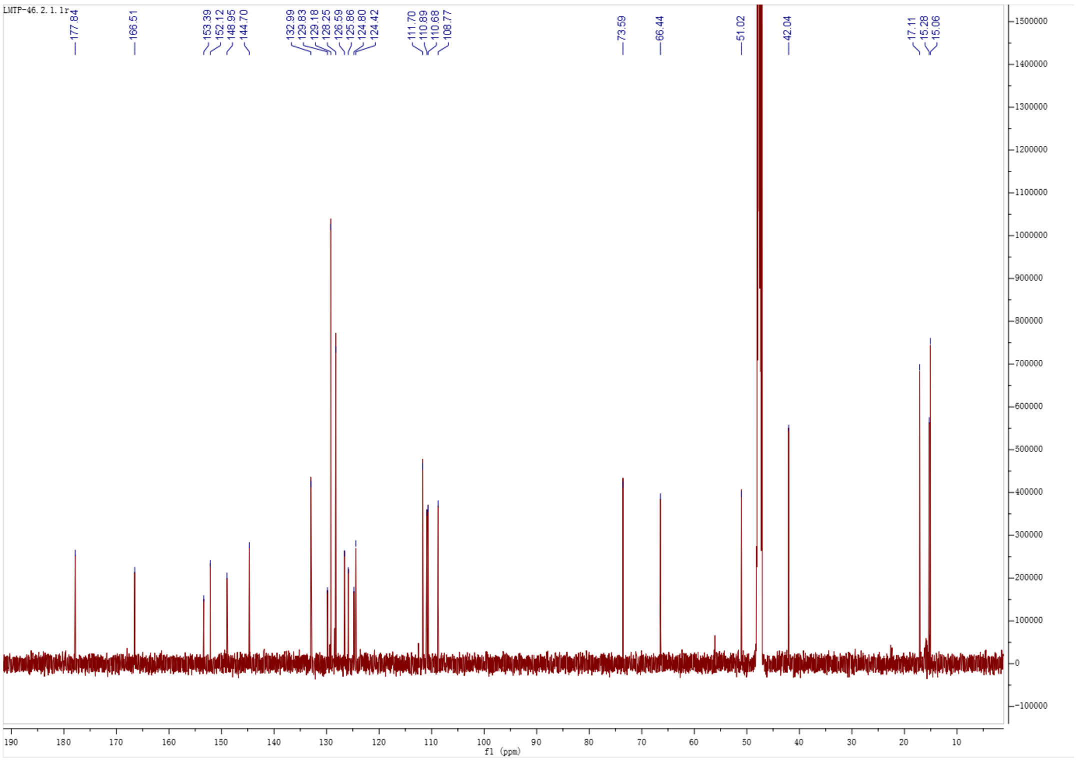

Figure S8. HSQC (600/150 MHz, Methanol-d_4) spectrum of paeobenzofuranone B (2).

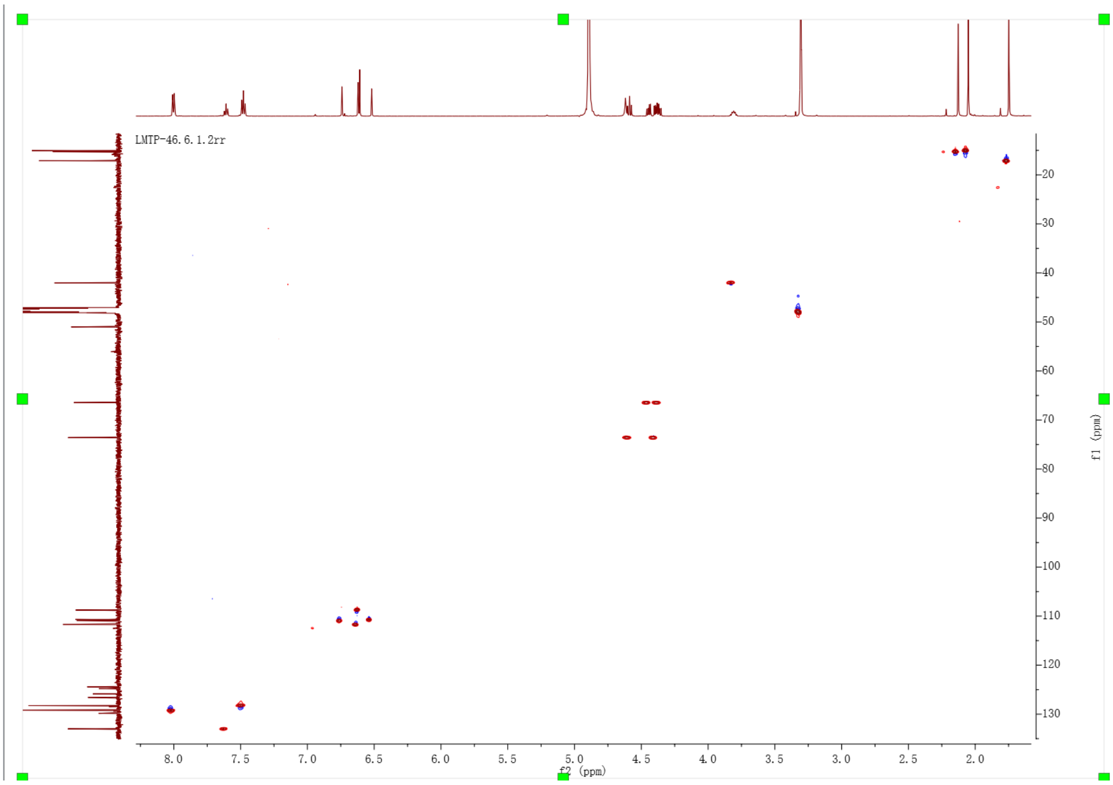

Figure S9. HMBC (600/150 MHz, Methanol-d_4) spectrum of paeobenzofuranone B (2).

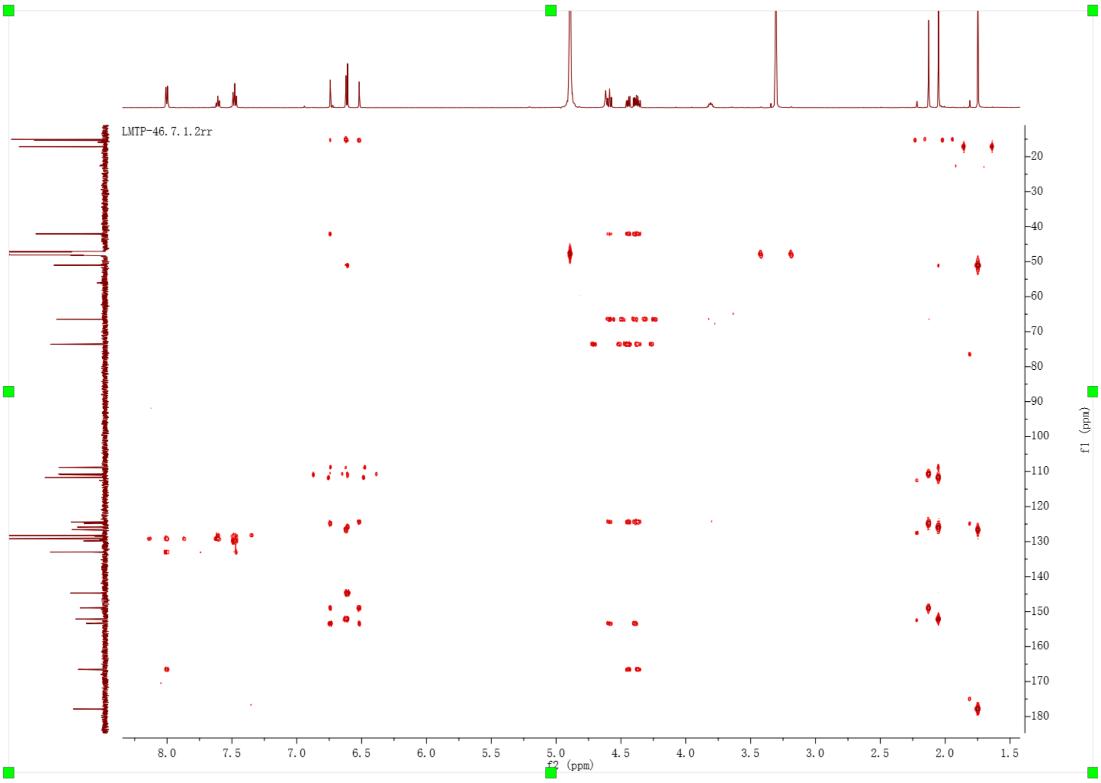

Figure S10. ^{1}H-^{1}H COSY (600/150 MHz, methanol-d_4) spectrum of paeobenzofuranone B (2).

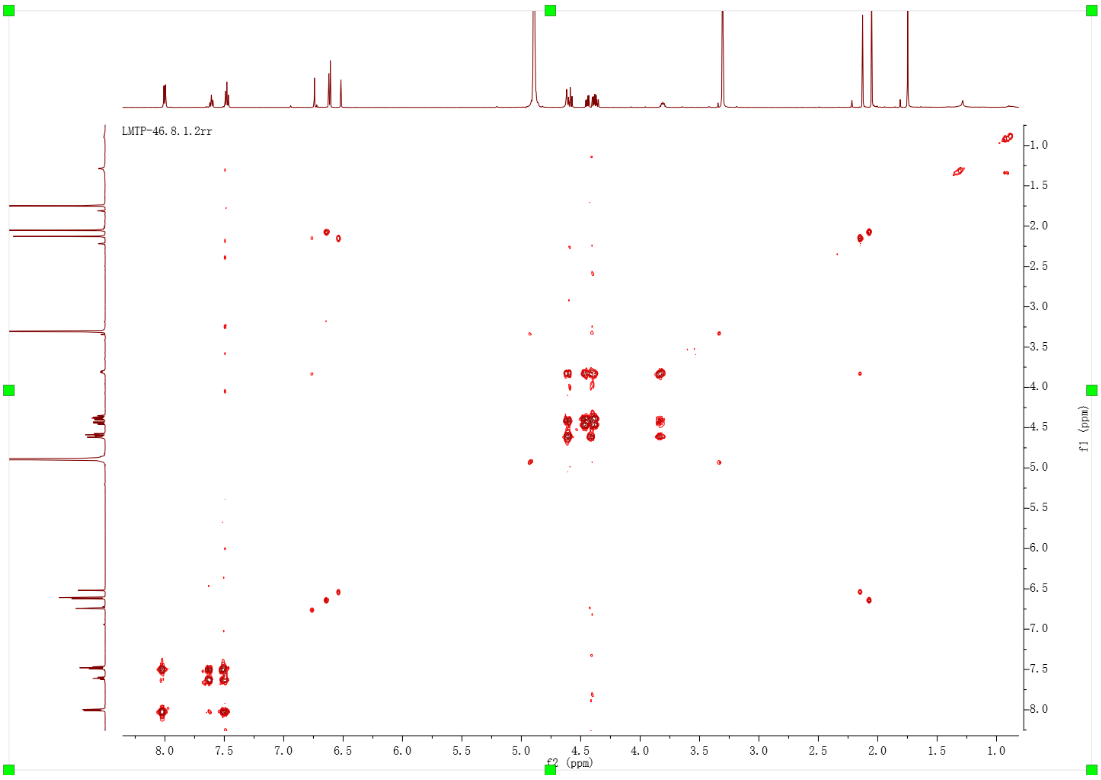

Figure S11. ROESY (600/150 MHz, methanol-d_4) spectrum of paeobenzofuranone B (2).

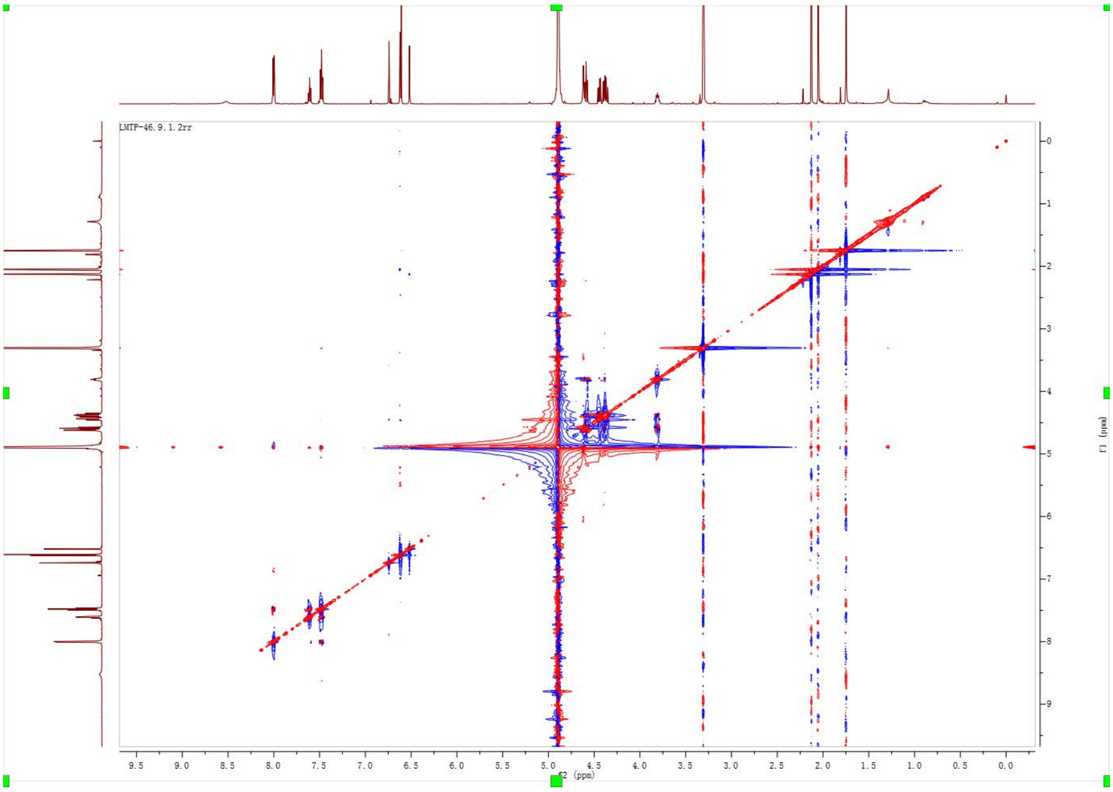

Figure S12. HRESIMS spectrum of paeobenzofuranone B (2)

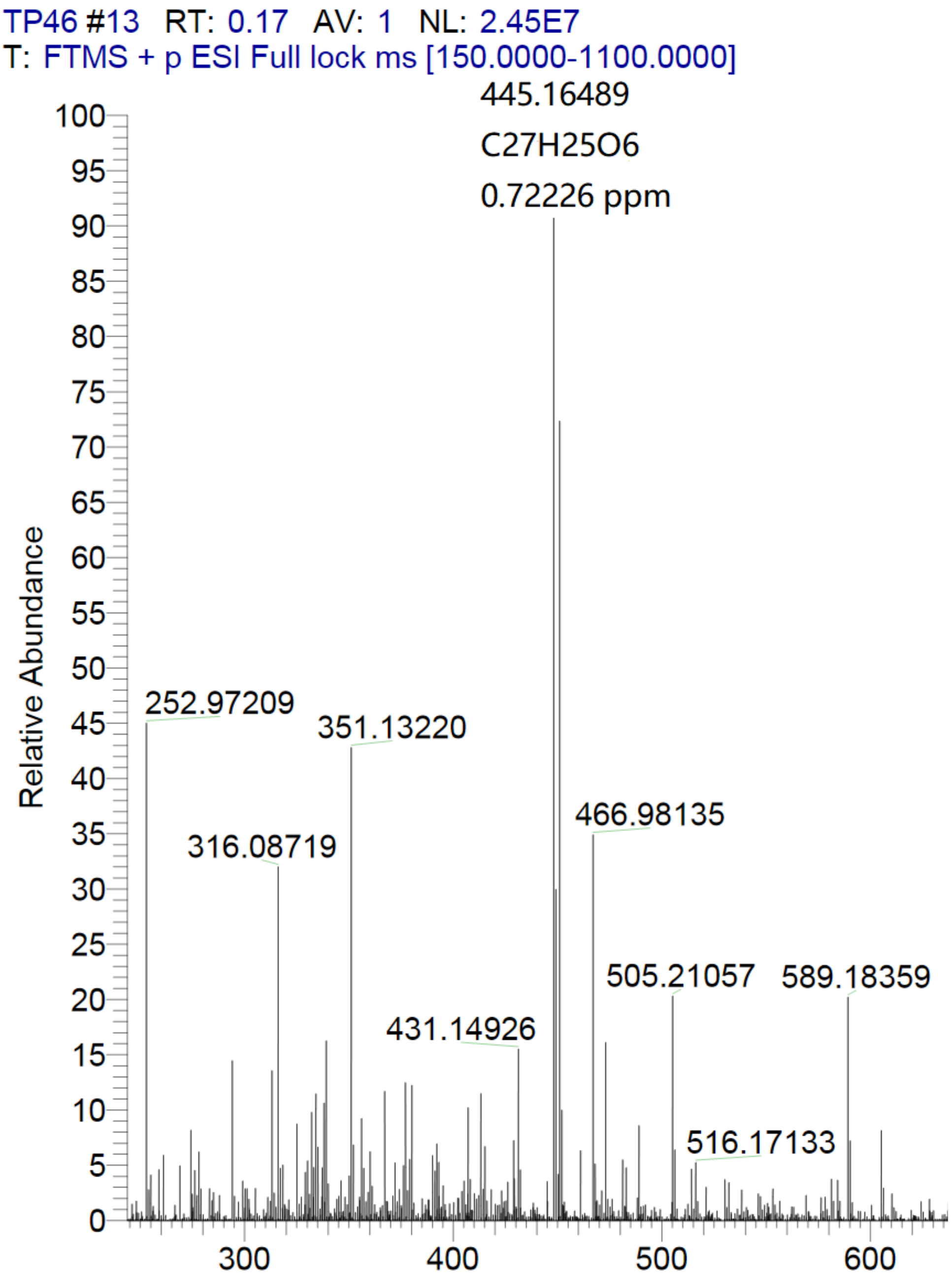

Figure S13. ^{1}**H NMR (600 MHz, Methanol-*d*$_4$) spectrum of paeobenzofuranone C (3).**

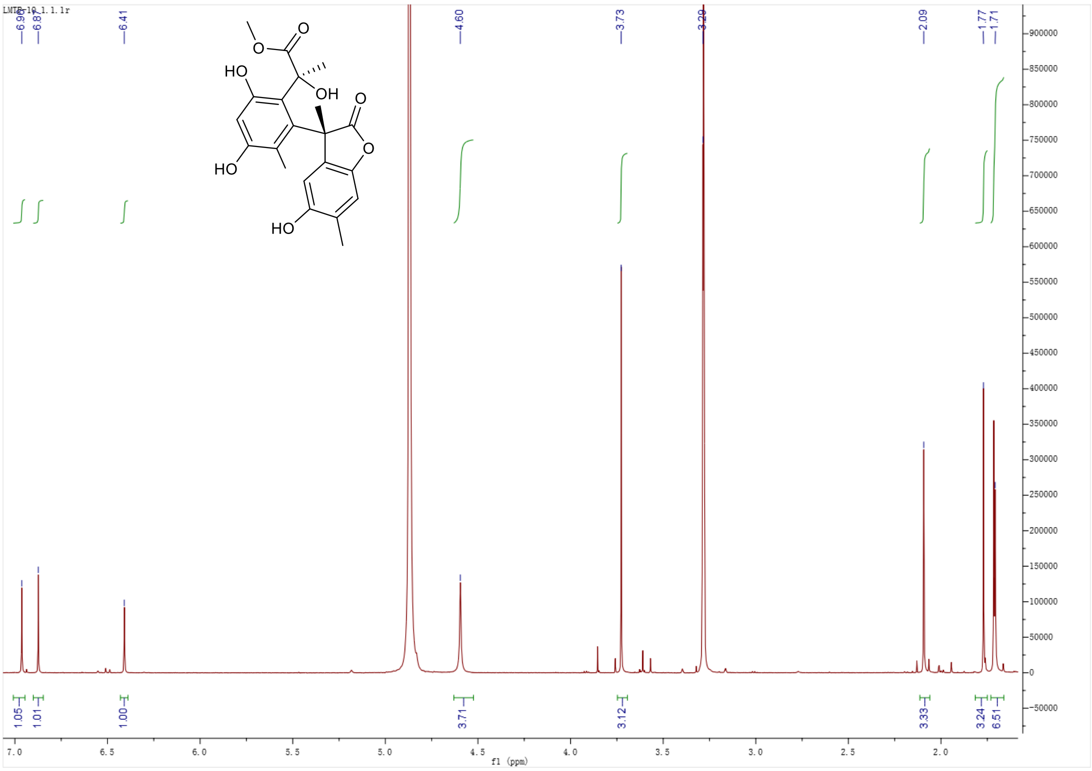

Figure S14. ^{13}**C NMR (150 MHz, Methanol-*d*$_4$) spectrum of paeobenzofuranone C (3).**

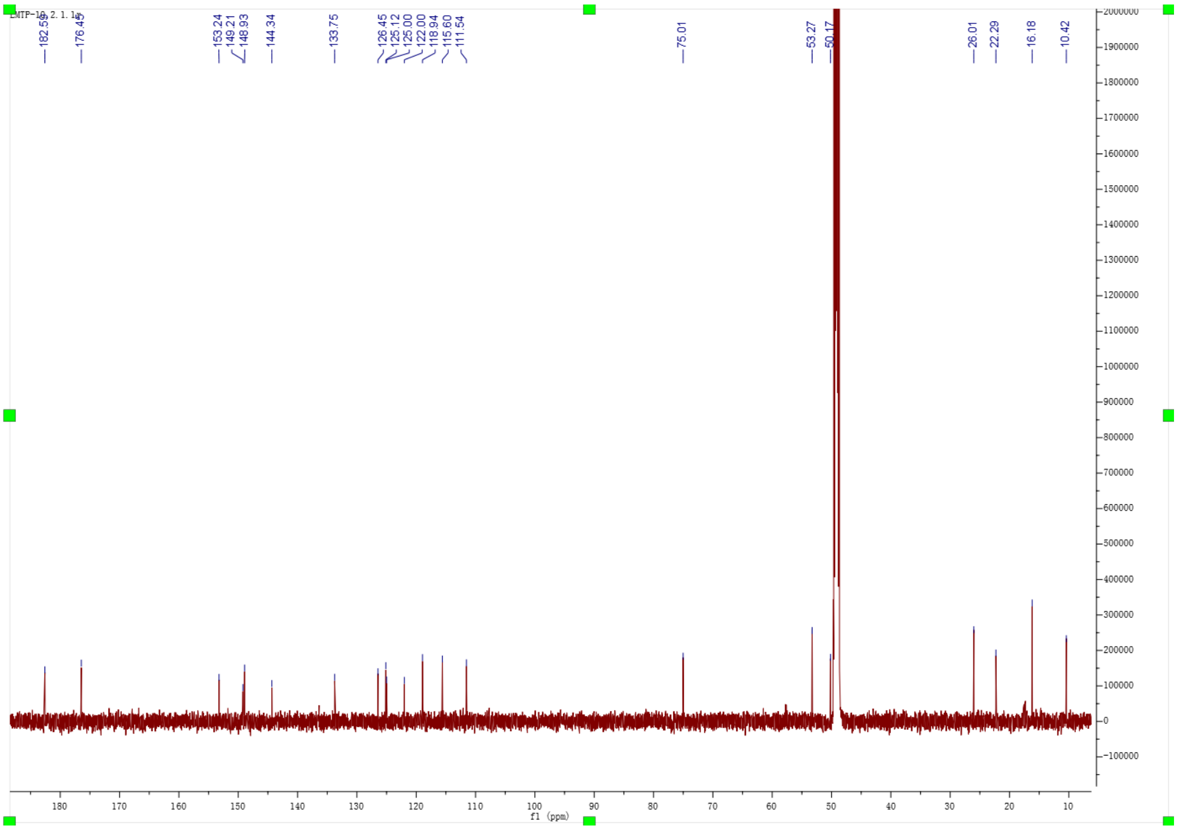

Figure S15. HSQC (600/150 MHz, Methanol-d_4) spectrum of paeobenzofuranone C (3).

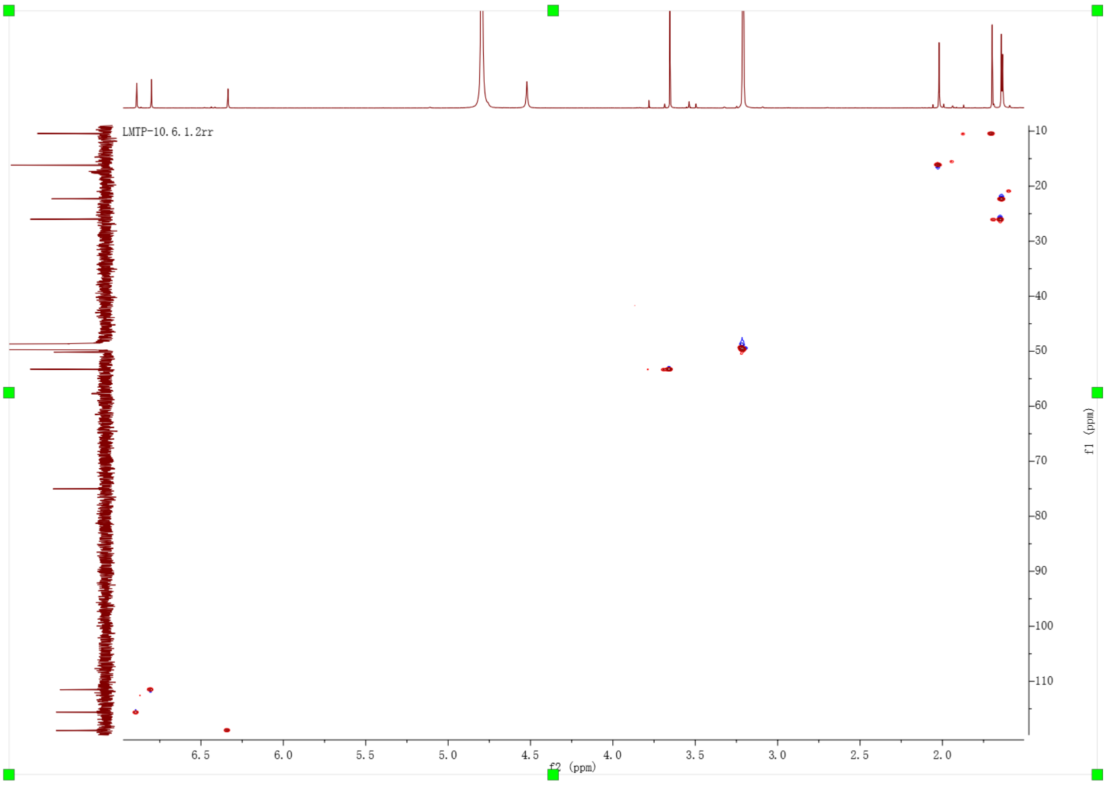

Figure S16. HMBC (600/150 MHz, Methanol-d_4) spectrum of paeobenzofuranone C (3).

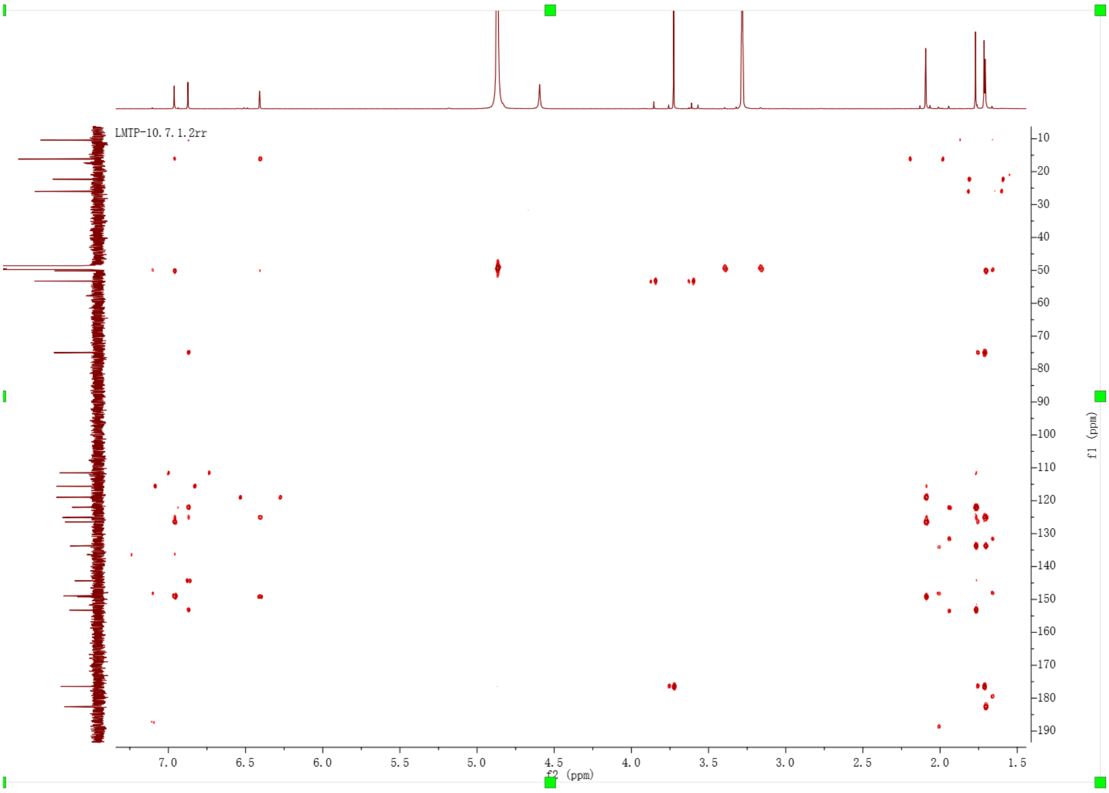

Figure S17. ^{1}H-^{1}H COSY (600 MHz, methanol-d_4) spectrum of paeobenzofuranone C (3).

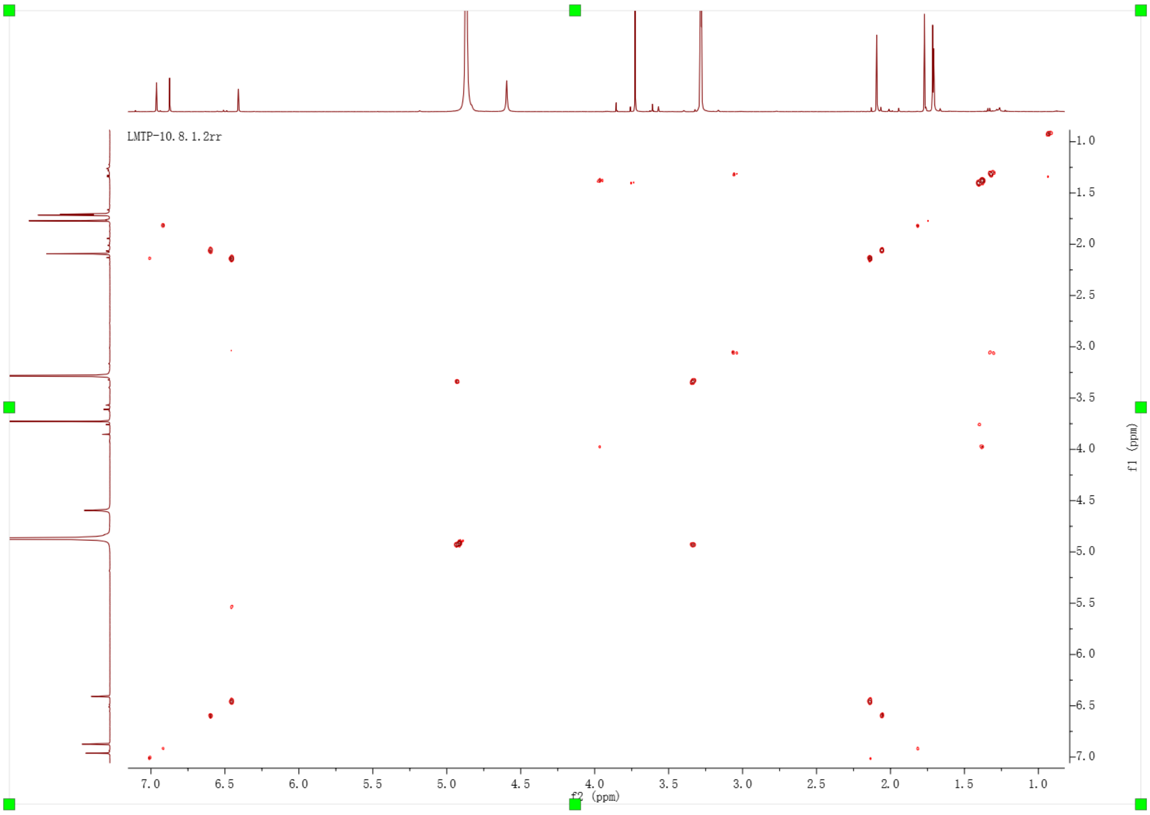

Figure S18. HRESIMS spectrum of paeobenzofuranone C (3)

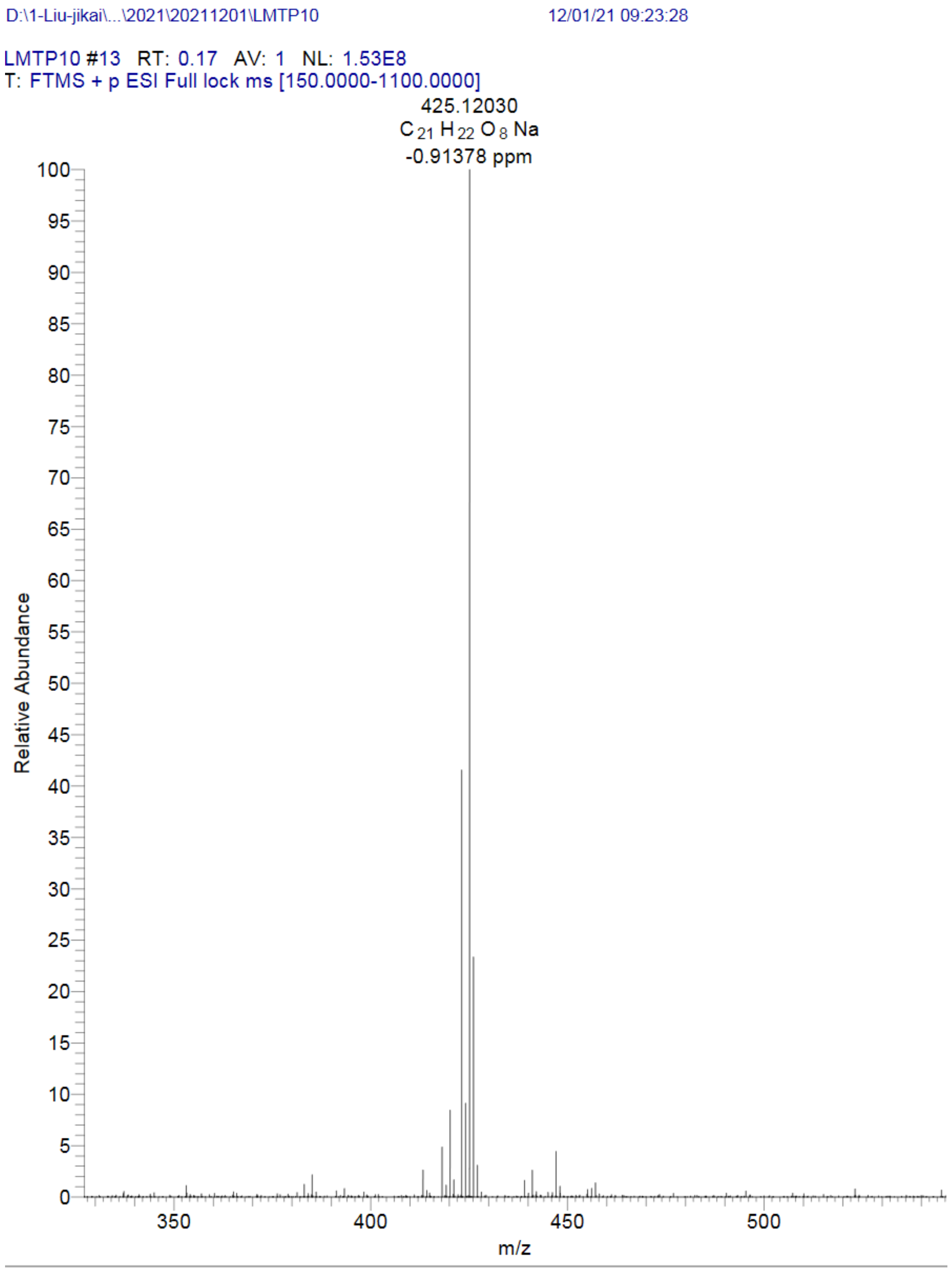

Figure S19. ¹H NMR (600 MHz, Methanol-d_4) spectrum of paeobenzofuranone D (4).

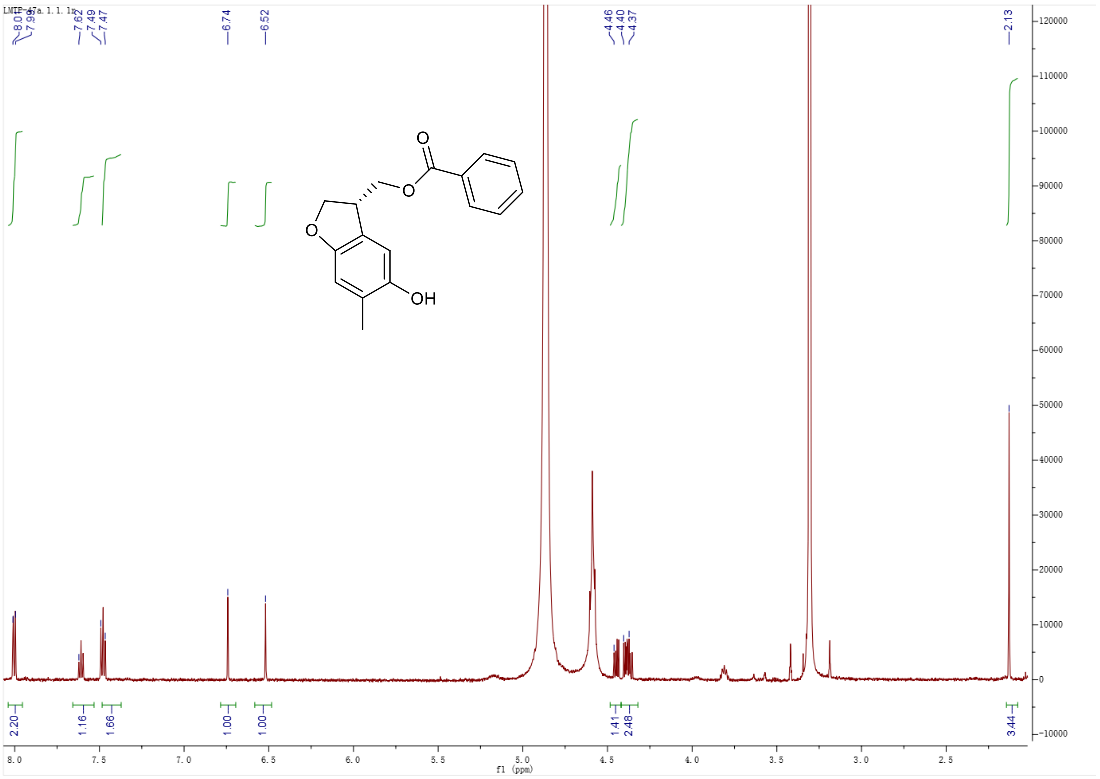

Figure S20. ¹³C NMR (150 MHz, Methanol-d_4) spectrum of paeobenzofuranone D (4).

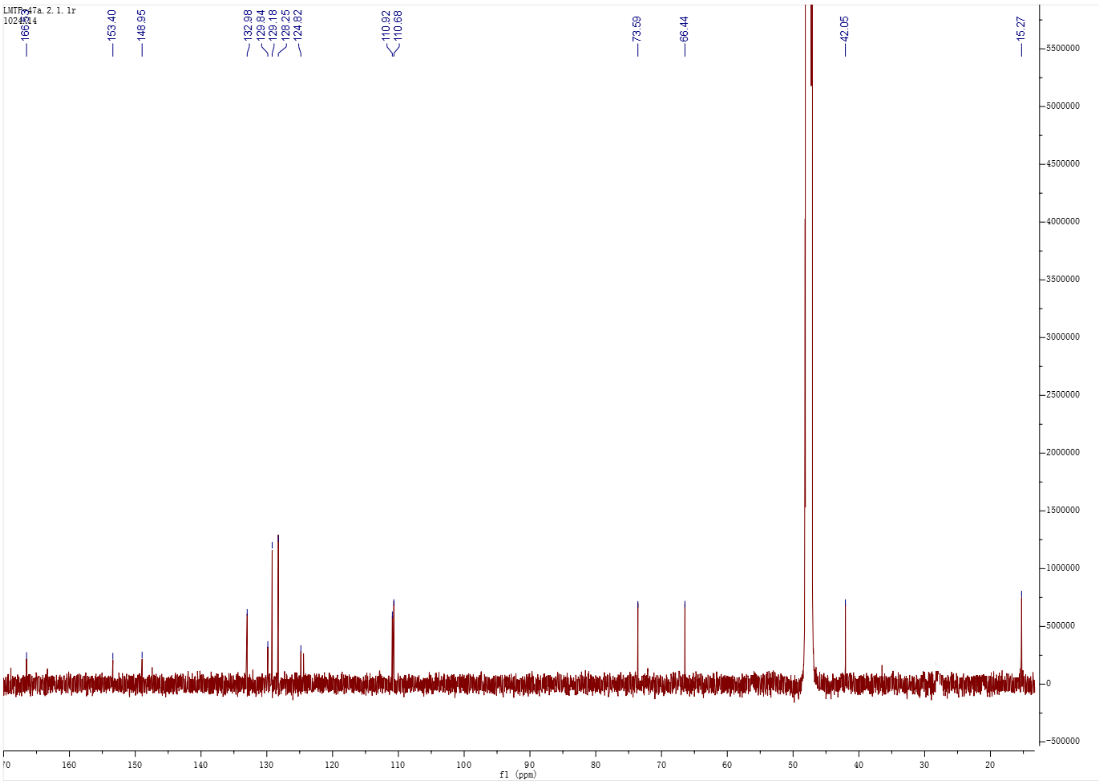

Figure S21. HSQC (600/150 MHz, Methanol-d_4) spectrum of paeobenzofuranone D (4).

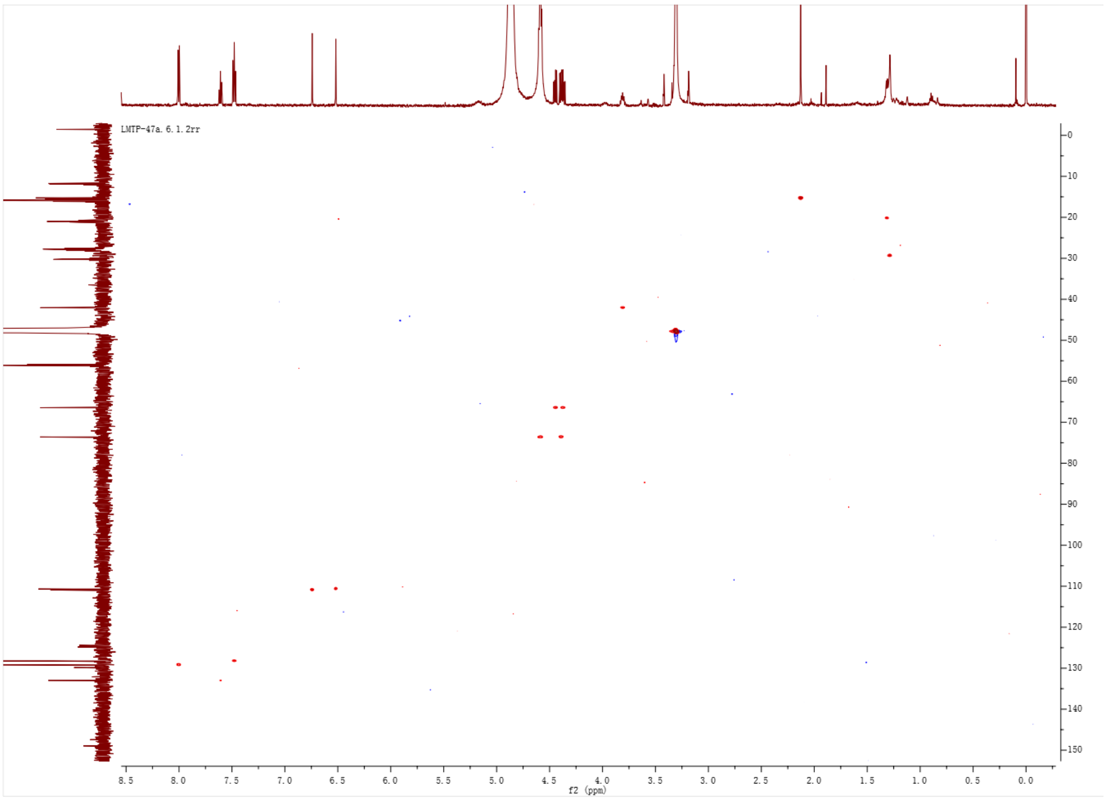

Figure S22. HMBC (600/150 MHz, Methanol-d_4) spectrum of paeobenzofuranone D (4).

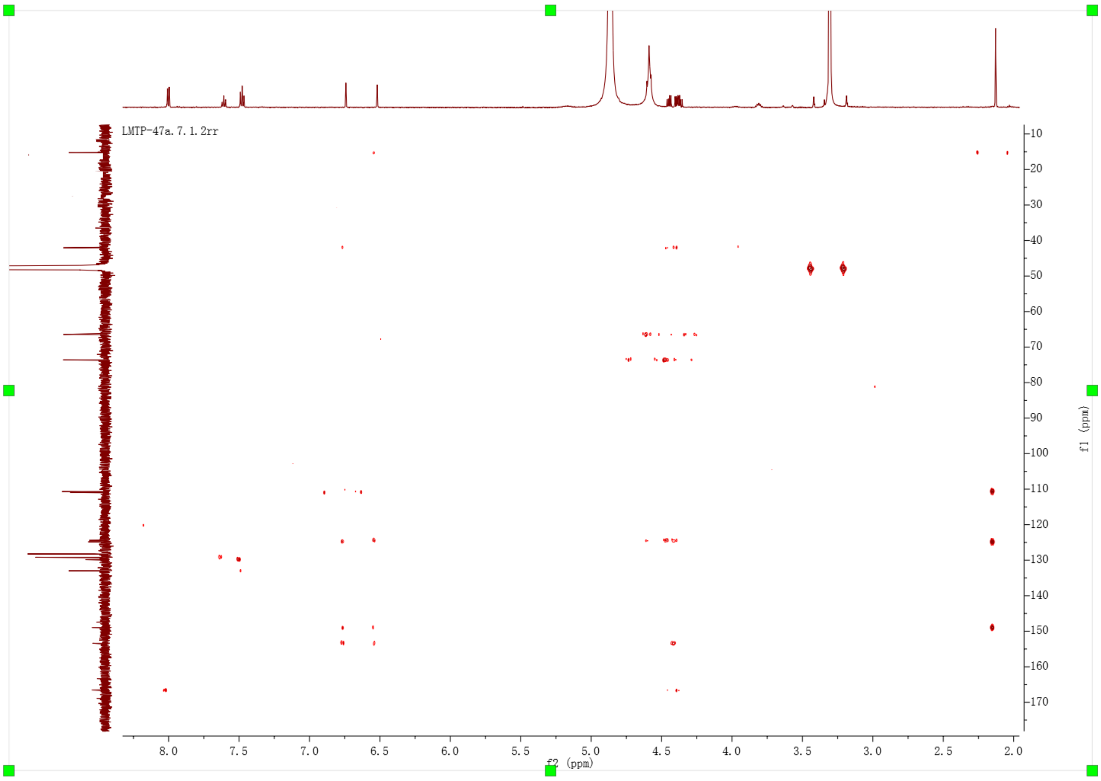

Figure S23. ^1H-^1H COSY (600, Methanol-d_4) spectrum of paeobenzofuranone D (4).

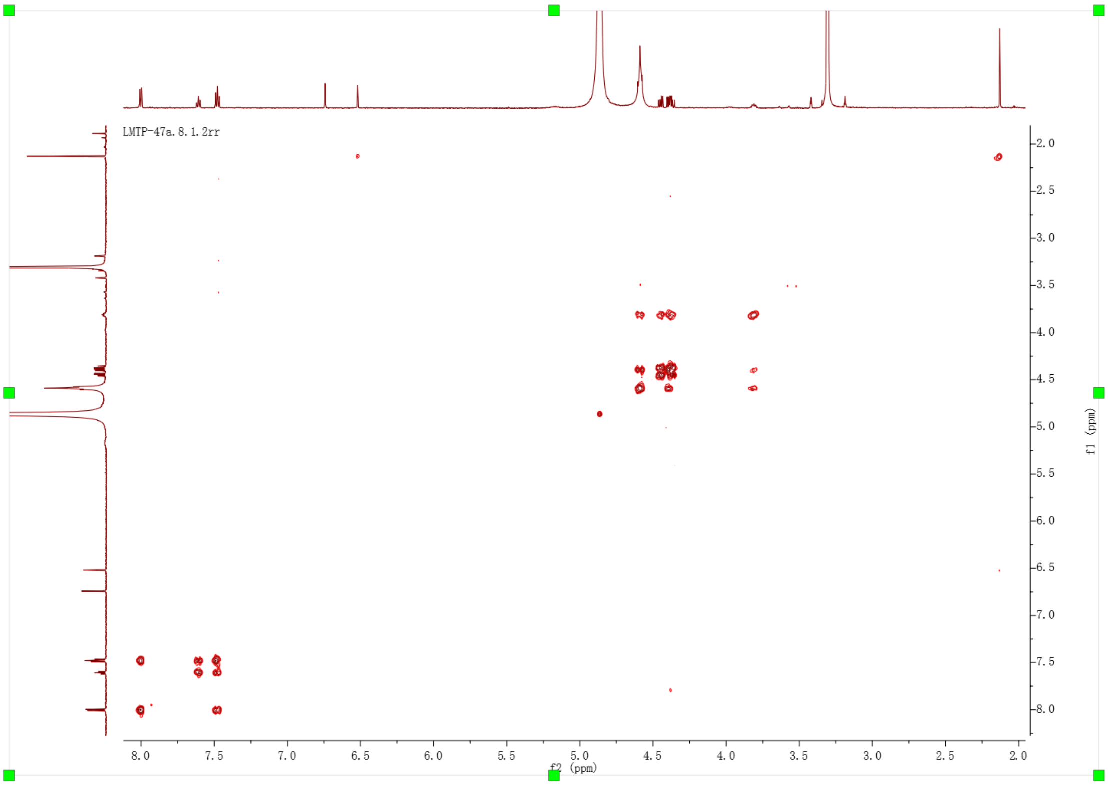

Figure S24. HRESIMS spectrum of paeobenzofuranone D (4)

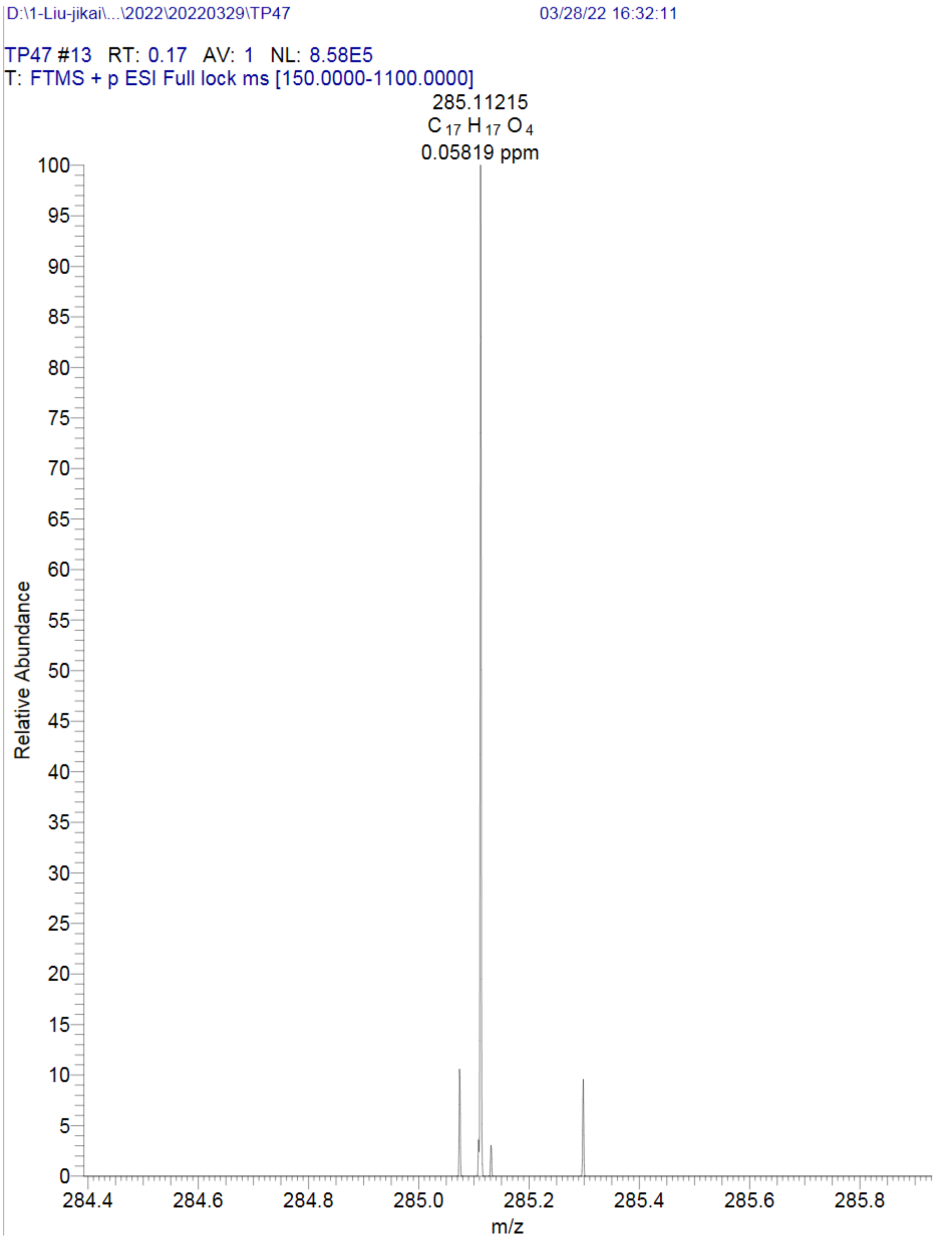

Figure S25. ^{1}**H NMR (600 MHz, Methanol-*d*$_4$) spectrum of paeobenzofuranone E (5).**

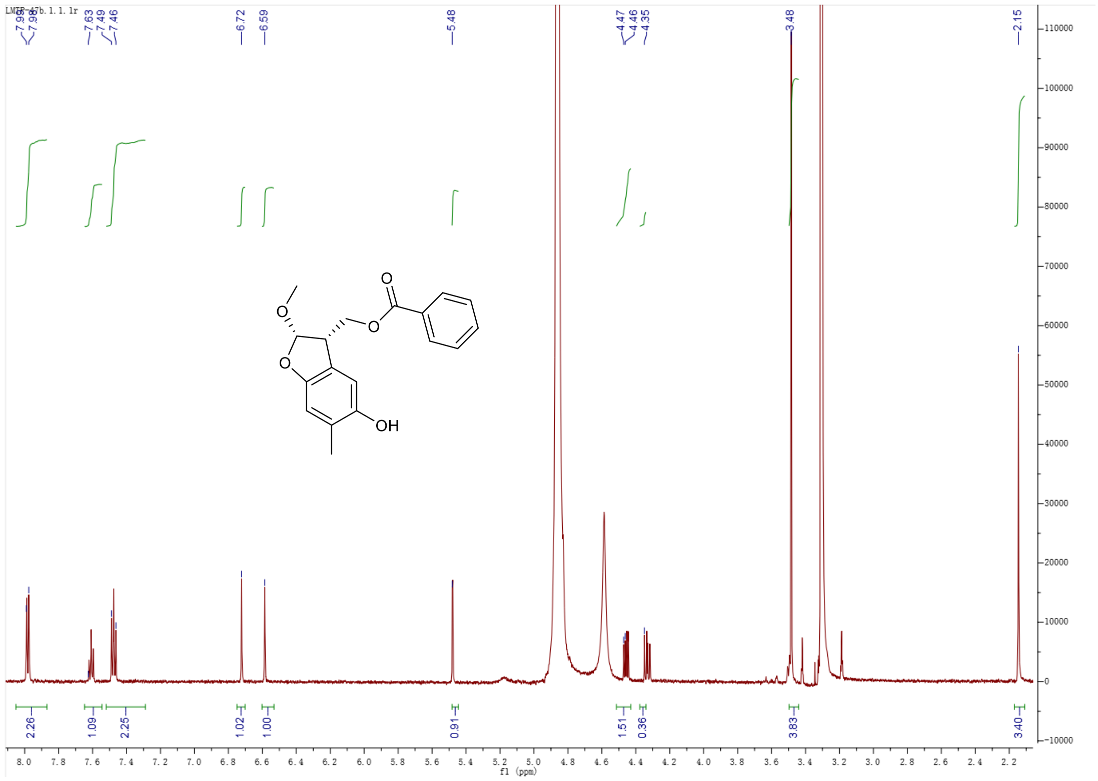

Figure S26. ^{13}**C NMR (150 MHz, Methanol-*d*$_4$) spectrum of paeobenzofuranone E (5).**

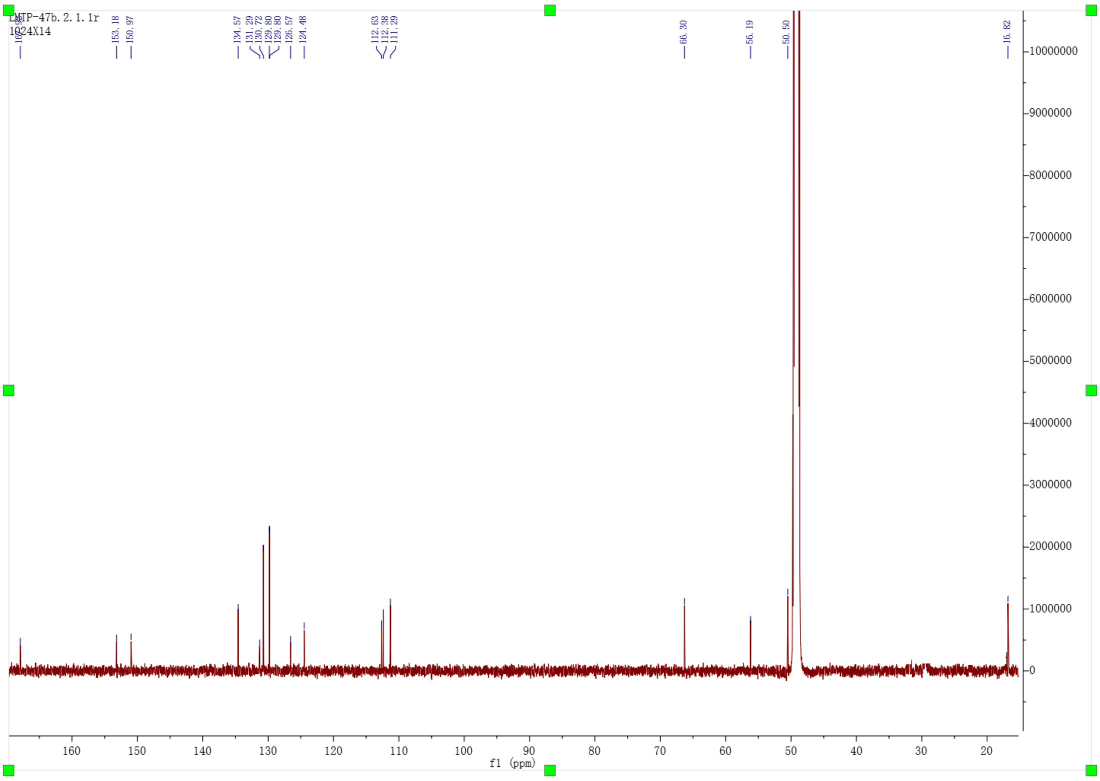

Figure S27. HSQC (600/150 MHz, Methanol-d_4) spectrum of paeobenzofuranone E (5).

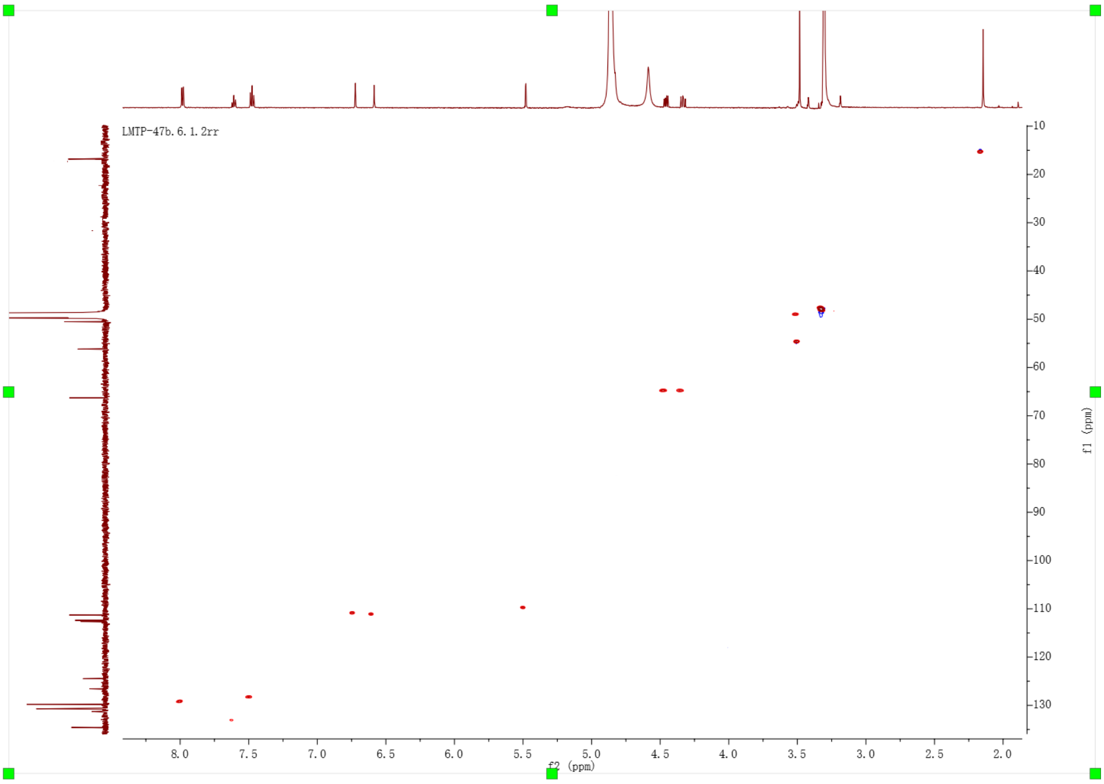

Figure S28. HMBC (600/150 MHz, Methanol-d_4) spectrum of paeobenzofuranone E (5).

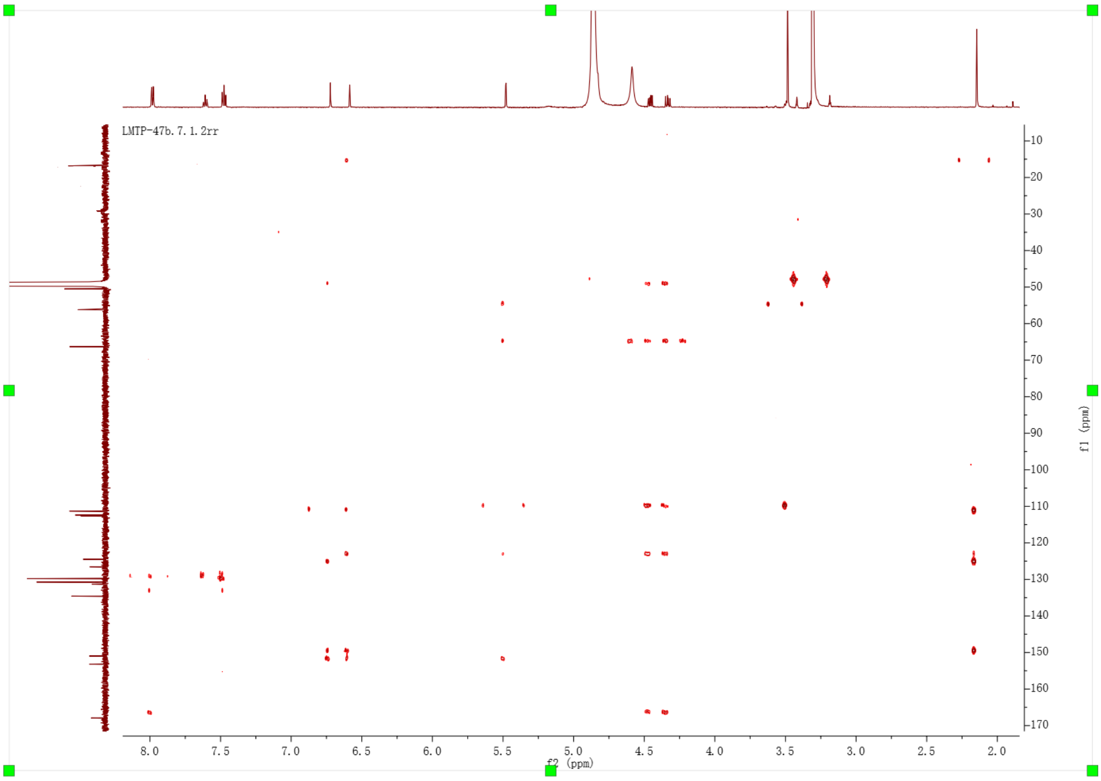

Figure S29. ^1H-^1H COSY (600, Methanol-d_4) spectrum of paeobenzofuranone E (5).

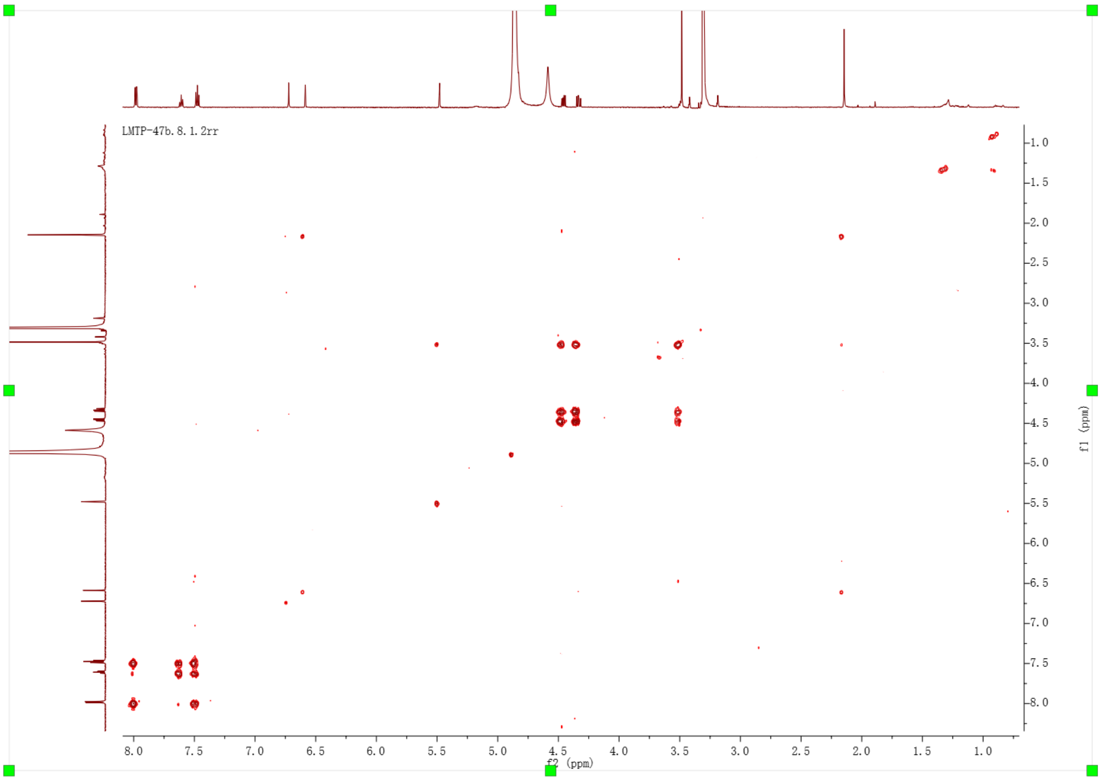

Figure S30. HRESIMS spectrum of paeobenzofuranone E (5)

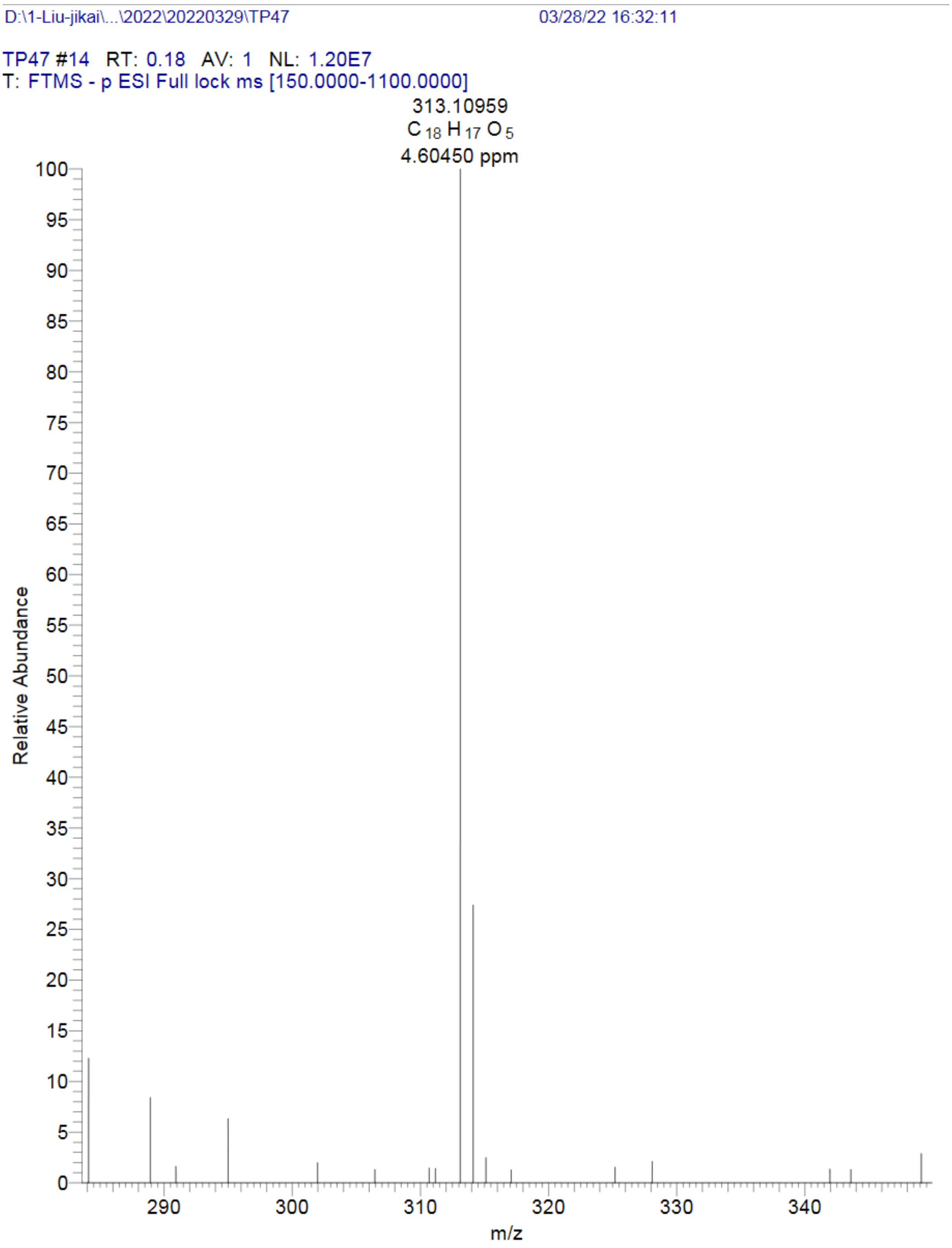

Section S2. Computational details for compounds 1–5.

Section S2-1. Computational details for paeobenzofuranone A (1) (ECD)

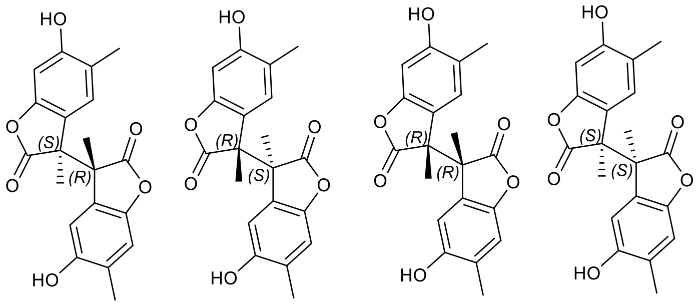

3*R*,3'*S*-**1a** 3*S*,3'*R*-**1b** 3*R*,3'*R*-**1c** 3*S*,3'*S*-**1d**

Conformation search based on molecular mechanics with MMFF force fields were performed for **1a**,**1b**,**1c** and **1d** gave 16 low-energy conformers with populations higher than 1%, respectively. All these conformers were further optimized by the density functional theory method at the B3LYP/6-311G(d) level by Gaussian 16 program package. The ECD were calculated using density functional theory (TDDFT) at B3LYP/6-31+G(d,p) level in methanol with IEFPCM model. The calculated ECD curves were all generated using SpecDis 1.71 with σ = 0.30 eV, and UV shift -15.7nm, respectively.

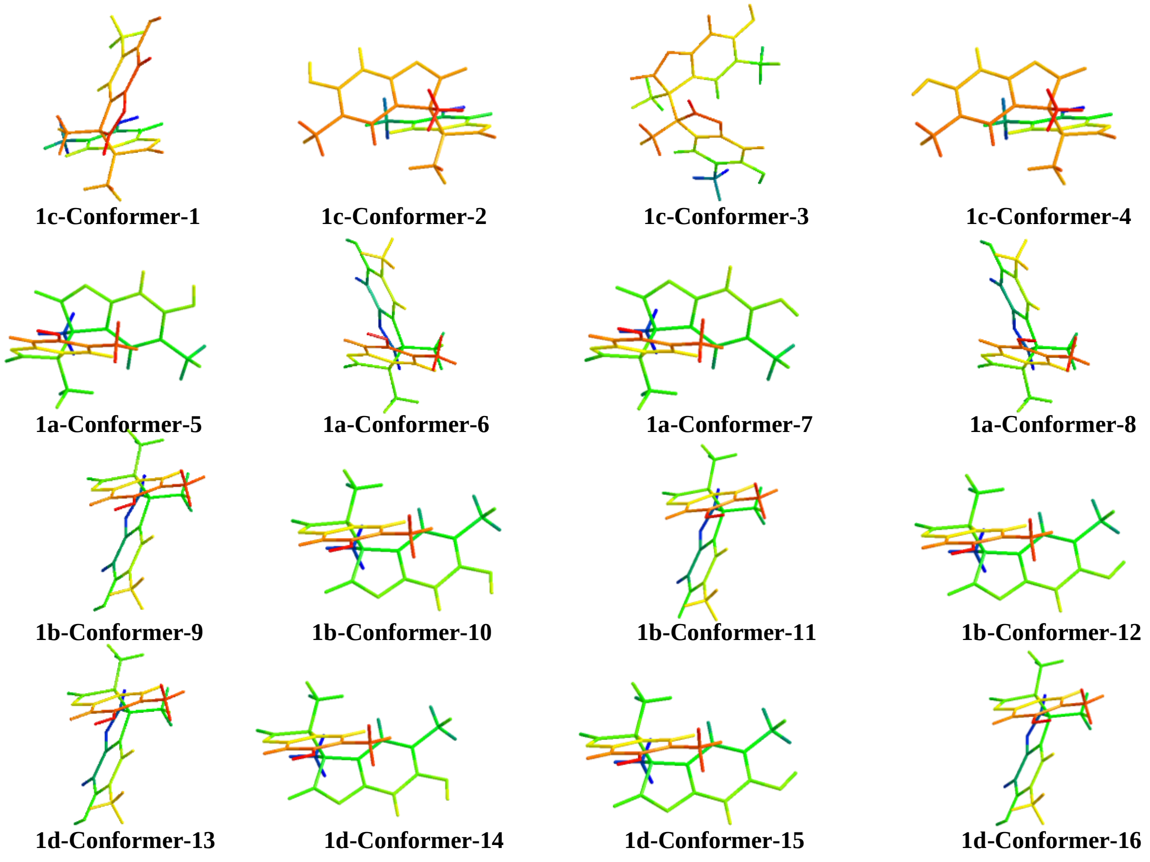

Figure S31. Low-energy Conformers of compound 1 in MeOH

Table S1. Energy analysis for conformers of **1** at B3LYP/6-31+G(d,p) level in the gas phase

Species	*E′=E+ZPE*	*E*	*H*	*G*	*ΔG*	*ΔE(kcal/mol)*	*PE%*
**1c-1**	-1224.096269	-1224.073042	-1224.072097	-1224.147276	0.000092	0.057731	19.77%
**1c-2**	-1224.096269	-1224.073042	-1224.072097	-1224.147276	0.000092	0.057731	19.77%
**1c-3**	-1224.095633	-1224.072406	-1224.071462	-1224.146579	0.000789	0.495105	9.45%
**1c-4**	-1224.095633	-1224.072406	-1224.071462	-1224.146579	0.000789	0.495105	9.45%
**1a-5**	-1224.096269	-1224.073042	-1224.072097	-1224.147276	0.000093	0.057741	19.97%
**1a-6**	-1224.096269	-1224.073042	-1224.072097	-1224.147276	0.000092	0.057731	19.77%
**1a-7**	-1224.095633	-1224.072406	-1224.071462	-1224.146579	0.000789	0.495105	0.00%
**1a-8**	-1224.095633	-1224.072406	-1224.071462	-1224.146579	0.000789	0.495105	0.00%
**1a-8**	-1224.096269	-1224.073042	-1224.072097	-1224.14727	0.000098	0.061496	0.00%
**1b-9**	-1224.096269	-1224.073042	-1224.072097	-1224.147276	0.000092	0.057731	0.00%
**1b-10**	-1224.095633	-1224.072406	-1224.071462	-1224.146579	0.000789	0.495105	0.00%
**1b-11**	-1224.095633	-1224.072406	-1224.071462	-1224.146579	0.000789	0.495105	0.00%
**1b-12**	-1224.095633	-1224.072406	-1224.071462	-1224.146579	0.000789	0.495105	0.00%
**1d-13**	-1224.096269	-1224.073042	-1224.072097	-1224.147276	0.000092	0.057731	0.00%
**1d-14**	-1224.096269	-1224.073042	-1224.072097	-1224.147276	0.000092	0.057731	0.00%
**1d-15**	-1224.095633	-1224.072406	-1224.071462	-1224.146579	0.000789	0.495105	0.00%
**1d-16**	-1224.095633	-1224.072406	-1224.071462	-1224.146579	0.000789	0.495105	0.00%

E, *E′*, *H*, *G*: total energy, total energy with zero point energy (ZPE), enthalpy, and Gibbs free energy

Section S2-2. Computational details for paeobenzofuranone B (2) (ECD)

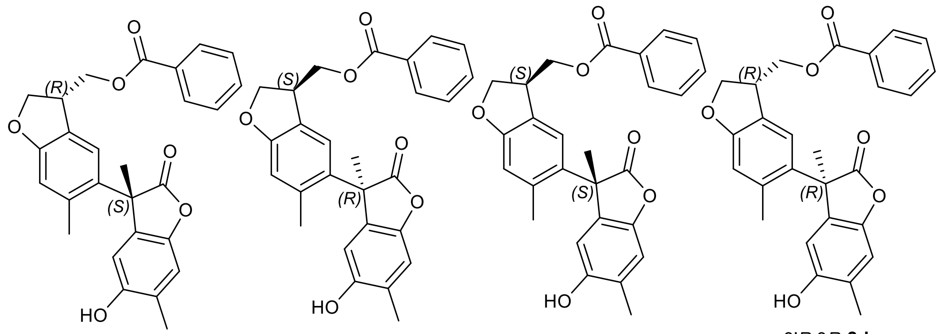

3'*S*,3*R*-**2a** 3'*R*,3*S*-**2b** 3'*S*,3*S*-**2c** 3'*R*,3*R*-**2d**

Conformation search based on molecular mechanics with MMFF force fields were performed for **2a**,**2b**,**1c** and **2d** gave 16 low-energy conformers with populations higher than 1%, respectively. All these conformers were further optimized by the density functional theory method at the B3LYP/6-311G(d) level by Gaussian 16 program package. The ECD were calculated using density functional theory (TDDFT) at B3LYP/6-31+G(d,p) level in methanol with IEFPCM model. The calculated ECD curves were all generated using SpecDis 1.71 with σ = 0.30 eV, and UV shift -32.8nm, respectively.

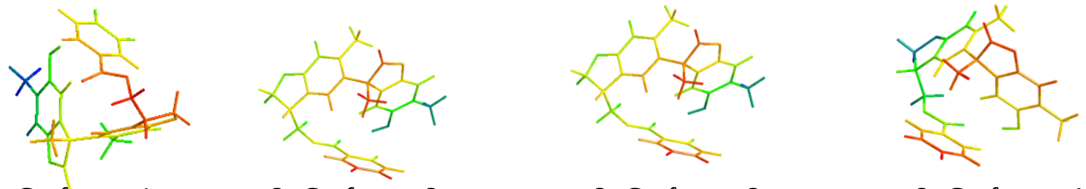

2c-Conformer-1 **2c-Conformer-2** **2c-Conformer-3** **2c-Conformer-4**

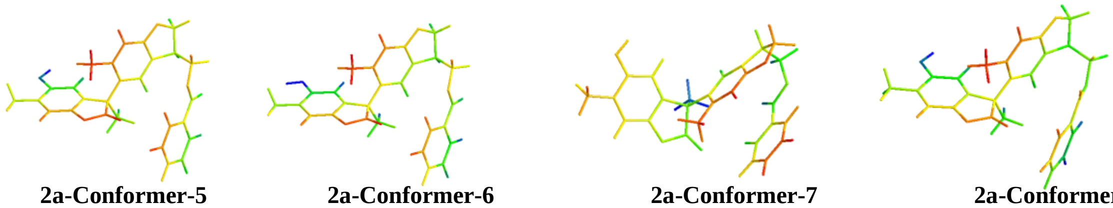

2a-Conformer-5 **2a-Conformer-6** **2a-Conformer-7** **2a-Conformer-8**

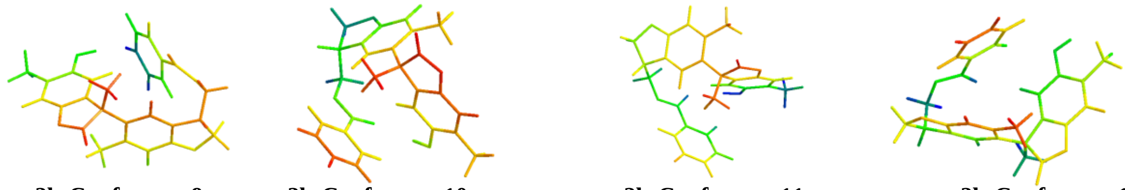

2b-Conformer-9 **2b-Conformer-10** **2b-Conformer-11** **2b-Conformer-12**

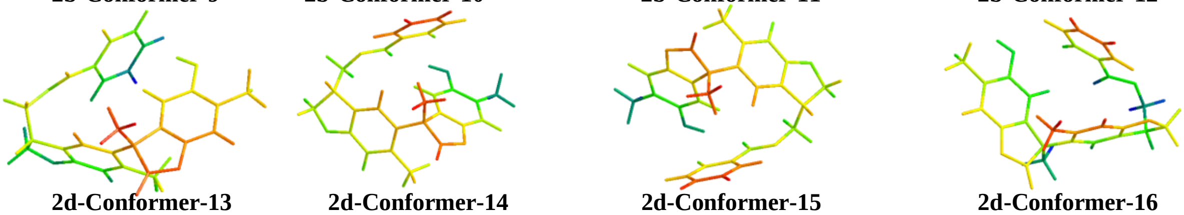

2d-Conformer-13 **2d-Conformer-14** **2d-Conformer-15** **2d-Conformer-16**

Figure S32. Low-energy Conformers of compound 2 in MeOH

Table S2. Energy analysis for conformers of **2** at B3LYP/6-31+G(d,p) level in the gas phase

Species	*E′=E+ZPE*	*E*	*H*	*G*	*ΔG*	*ΔE(kcal/mol)*	*PE%*
**2c-1**	-1494.382728	-1494.354411	-1494.353466	-1494.442059	0.000001	0.000628	90.69%
**2c-2**	-1494.381002	-1494.352961	-1494.352017	-1494.4389	0.003160	1.982930	3.19%
**2c-3**	-1494.381002	-1494.352961	-1494.352017	-1494.438899	0.003161	1.983558	3.18%
**2c-4**	-1494.381043	-1494.352921	-1494.351977	-1494.438785	0.003275	2.055094	2.82%
**2a-5**	-1494.376179	-1494.347885	-1494.346941	-1494.435446	0.006614	4.150348	0.08%
**2a-6**	-1494.375493	-1494.347182	-1494.346238	-1494.434685	0.007375	4.627883	0.04%
**2a-7**	-1494.378155	-1494.349974	-1494.349029	-1494.436381	0.005679	3.563626	0.03%
**2a-8**	-1494.375563	-1494.347255	-1494.34631	-1494.434251	0.007809	4.900222	0.06%
**2b-9**	-1494.382728	-1494.354411	-1494.353466	-1494.442059	0.000001	0.000628	0.10%
**2b-10**	-1494.381002	-1494.352961	-1494.352017	-1494.4389	0.003160	1.982930	0.05%
**2b-11**	-1494.381002	-1494.352961	-1494.352017	-1494.438899	0.003161	1.983558	0.06%
**2b-12**	-1494.381043	-1494.352921	-1494.351977	-1494.438785	0.003275	2.055094	0.12%
**2d-13**	-1494.382729	-1494.354411	-1494.353467	-1494.44206	0.000000	0.000000	0.13%
**2d-14**	-1494.381002	-1494.352961	-1494.352017	-1494.438901	0.003159	1.982303	0.07%
**2d-15**	-1494.381002	-1494.352961	-1494.352017	-1494.4389	0.003160	1.982930	0.08%
**2d-16**	-1494.381043	-1494.352921	-1494.351977	-1494.438784	0.003276	2.055721	0.09%

E, *E′*, *H*, *G*: total energy, total energy with zero point energy (ZPE), enthalpy, and Gibbs free energy

Section S2-3. Computational details for paeobenzofuranone C (3) (ECD)

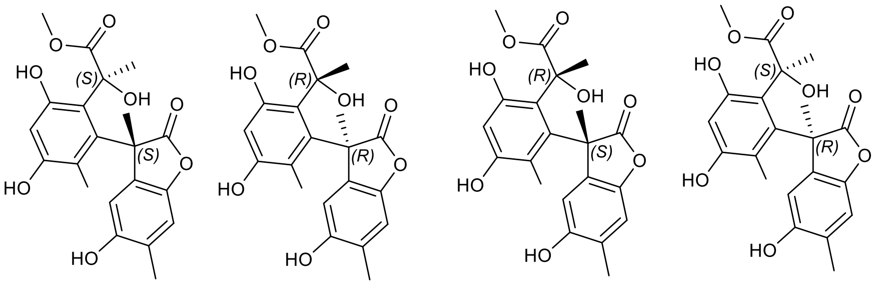

3*S*,7'*S*-**3a** 3*R*,7'*R*-**3b** 3*S*,7'*R*-**3c** 3*R*,7'*S*-**3d**

Conformation search based on molecular mechanics with MMFF force fields were performed for **3a**,**3b**,**3c** and **3d** gave 16 low-energy conformers with populations higher than 1%, respectively. All these conformers were further optimized by the density functional theory method at the B3LYP/6-311G(d) level by Gaussian 16 program package. The ECD were calculated using density functional theory (TDDFT) at B3LYP/6-31+G(d,p) level in methanol with IEFPCM model. The calculated ECD curves were all generated using SpecDis 1.71 with σ = 0.30 eV, and UV shift -27.5nm, respectively.

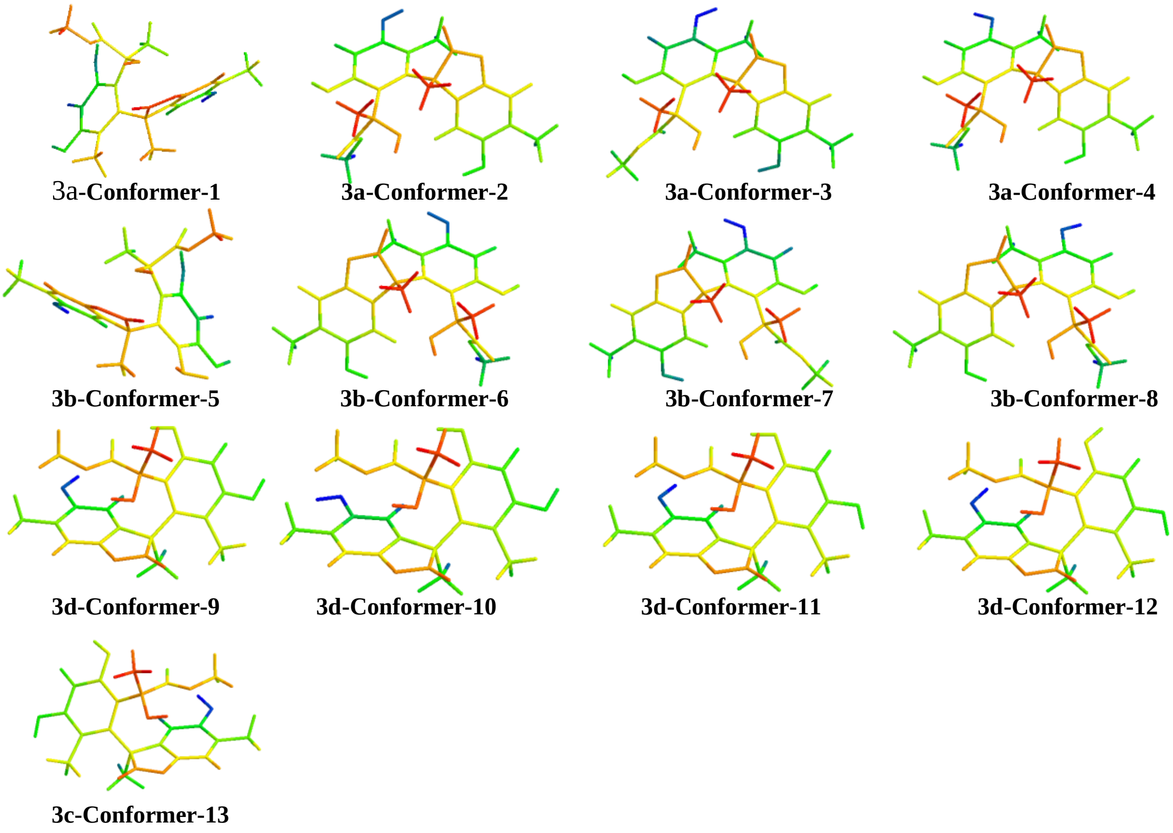

Figure S33. Low-energy Conformers of compound 3 in MeOH

Table S3. Energy analysis for conformers of **3** at B3LYP/6-31+G(d,p) level in the gas phase

Species	*E′=E+ZPE*	*E*	*H*	*G*	*ΔG*	*ΔE(kcal/mol)*	*PE%*
**3a-1**	-1413.275394	-1413.248319	-1413.247374	-1413.3301	0.012476	7.828809	34.50%
**3a-2**	-1413.27436	-1413.246906	-1413.245961	-1413.329161	0.013415	8.418040	12.75%
**3a-3**	-1413.271933	-1413.244367	-1413.243423	-1413.327278	0.015298	9.599640	1.73%
**3a-4**	-1413.273028	-1413.245435	-1413.244491	-1413.32802	0.014556	9.134028	3.81%
**3b-5**	-1413.275395	-1413.24832	-1413.247376	-1413.330099	0.012477	7.829436	34.46%
**3b-6**	-1413.27436	-1413.246906	-1413.245961	-1413.329161	0.013415	8.418040	12.75%
**3b-7**	-1413.27436	-1413.246906	-1413.245961	-1413.329161	0.013415	8.418040	0.06%
**3d-8**	-1413.273028	-1413.245435	-1413.244491	-1413.32802	0.014556	9.134028	0.08%
**3d-9**	-1413.29006	-1413.26338	-1413.262436	-1413.342576	0.000000	0.000000	0.10%
**3d-10**	-1413.287228	-1413.260494	-1413.25955	-1413.339761	0.002815	1.766439	0.03%
**3d-11**	-1413.289106	-1413.262454	-1413.26151	-1413.341566	0.001010	0.633785	0.02%
**3d-12**	-1413.279469	-1413.252272	-1413.251328	-1413.333047	0.009529	5.979538	0.04%
**3c-13**	-1413.29006	-1413.26338	-1413.262436	-1413.342576	0.000000	0.000000	0.09%

E, *E′*, *H*, *G*: total energy, total energy with zero point energy (ZPE), enthalpy, and Gibbs free energy

Section S2-4. Computational details for paeobenzofuranone D (4) (ECD)

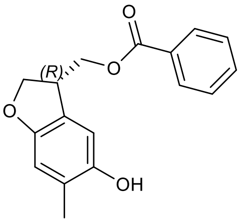

3*R*-**4a**

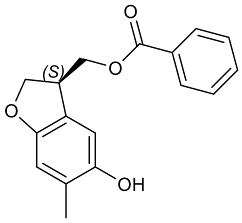

3*S*-**4b**

Conformation search based on molecular mechanics with MMFF force fields were performed for **4a**,**4b**,**4c** and **4d** gave 7 low-energy conformers with populations higher than 1%, respectively. All these conformers were further optimized by the density functional theory method at the B3LYP/6-311G(d) level by Gaussian 16 program package. The ECD were calculated using density functional theory (TDDFT) at B3LYP/6-31+G(d,p) level in methanol with IEFPCM model. The calculated ECD curves were all generated using SpecDis 1.71 with σ = 0.30 eV, and UV shift -25.8nm, respectively.

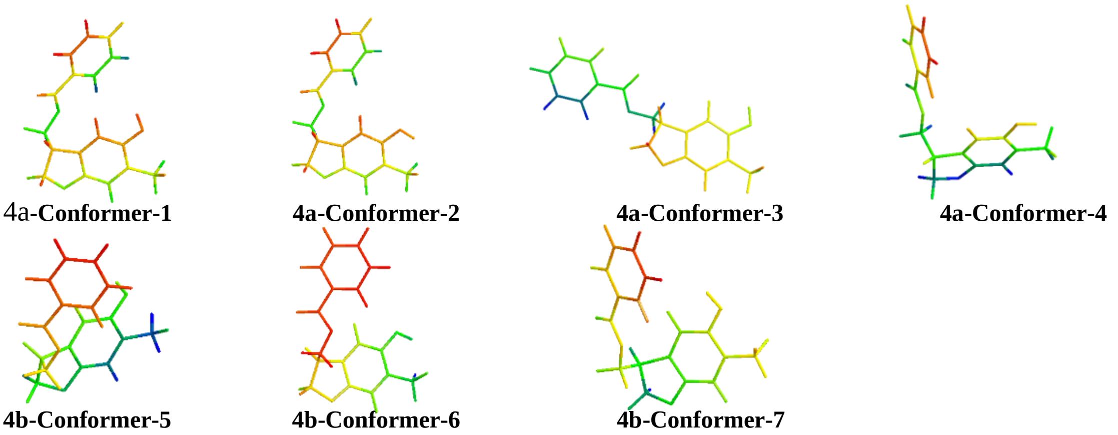

Figure S34. Low-energy Conformers of compound 4 in MeOH

Table S4. Energy analysis for conformers of **4** at B3LYP/6-31+G(d,p) level in the gas phase

Species	*E'=E+ZPE*	*E*	*H*	*G*	*ΔG*	*ΔE(kcal/mol)*	*PE%*
**4a-1**	-956.960332	-956.942475	-956.941531	-957.00803	0. 000001	0. 000628	27. 95%
**4a-2**	-956.959011	-956.940959	-956.940015	-957.007156	0. 000875	0. 549071	11. 07%
**4a-3**	-956.959753	-956.941839	-956.940895	-957.007608	0. 000423	0. 265437	17. 87%
**4a-4**	-956.957372	-956.939175	-956.93823	-957.006208	0. 001823	1. 143950	4. 05%
**4b-5**	-956.960332	-956.942475	-956.94153	-957.008031	0. 000000	0. 000000	27. 98%
**4b-6**	-956.959011	-956.940959	-956.940015	-957.007156	0. 000875	0. 549071	11. 07%
**4b-7**	-956.960332	-956.942475	-956.94153	-957.00803	0. 000001	0. 000628	0. 00%

E, *E'*, *H*, *G*: total energy, total energy with zero point energy (ZPE), enthalpy, and Gibbs free energy

Section S2-5. Computational details for paeobenzofuranone E (5) (ECD)

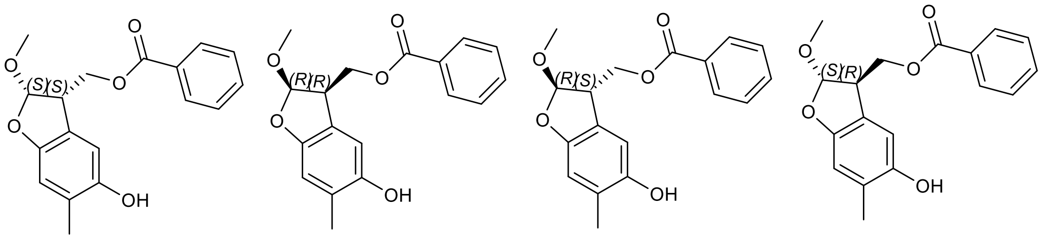

2*S*,3*S*-**5a** 2*R*,3*R*-**5b** 2*R*,3*S*-**5c** 2*S*,3*R*-**5d**

Conformation search based on molecular mechanics with MMFF force fields were performed for **5a**,**5b**,**5c** and **5d** gave 15 low-energy conformers with populations higher than 1%, respectively. All these conformers were further optimized by the density functional theory method at the B3LYP/6-311G(d) level by Gaussian 16 program package. The ECD were calculated using density functional theory (TDDFT) at B3LYP/6-31+G(d,p) level in methanol with IEFPCM model. The calculated ECD curves were all generated using SpecDis 1.71 with σ = 0.30 eV, and UV shift -28.6nm, respectively.

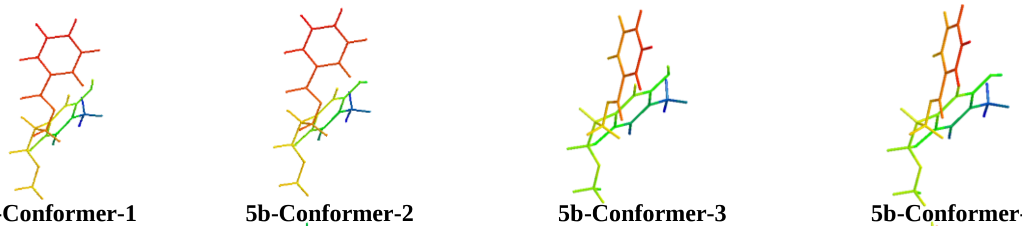

5b-**Conformer-1** **5b-Conformer-2** **5b-Conformer-3** **5b-Conformer-4**

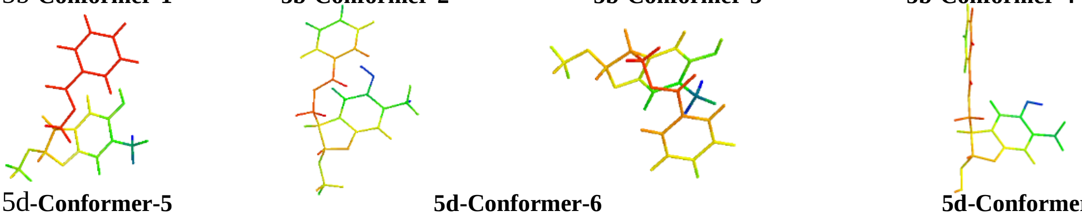

5d-**Conformer-5** **5d-Conformer-6** **5d-Conformer-7**
5d-Conformer-8

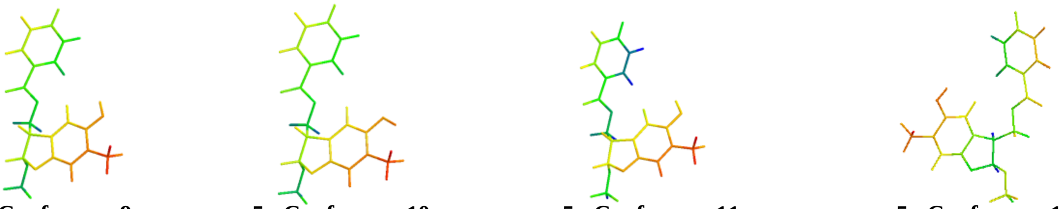

5c-Conformer-9 **5c-Conformer-10** **5c-Conformer-11** **5c-Conformer-12**

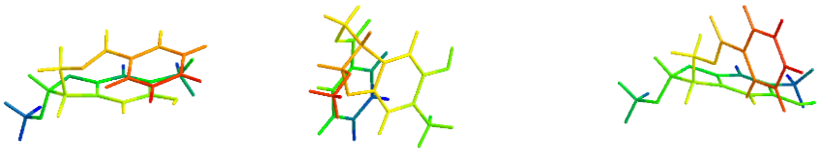

5a-Conformer-13 **5a-Conformer-14** **5a-Conformer-15**

Figure S35. Low-energy Conformers of compound 5 in MeOH

Table S5. Energy analysis for conformers of **5** at B3LYP/6-31+G(d,p) level in the gas phase

Species	*E′=E+ZPE*	*E*	*H*	*G*	*ΔG*	*ΔE(kcal/mol)*	*PE%*
**5b-1**	-1071.328153	-1071.30774	-1071.306796	-1071.379079	0.001836	1.152107	29.80%
**5b-2**	-1071.327025	-1071.306577	-1071.305633	-1071.377936	0.002979	1.869351	8.87%
**5b-3**	-1071.327364	-1071.306777	-1071.305833	-1071.378811	0.002104	1.320280	22.43%
**5b-4**	-1071.326143	-1071.305465	-1071.304521	-1071.377568	0.003347	2.100274	6.01%
**5d-5**	-1071.327342	-1071.306839	-1071.305895	-1071.378945	0.001970	1.236194	25.85%
**5d-6**	-1071.327765	-1071.307473	-1071.306529	-1071.377717	0.003198	2.006775	7.04%
**5d-7**	-1071.33102	-1071.310739	-1071.309795	-1071.380915	0.000000	0.000000	0.03%
**5d-8**	-1071.324986	-1071.304157	-1071.303213	-1071.377435	0.003480	2.183733	0.01%
**5c-9**	-1071.328153	-1071.30774	-1071.306796	-1071.379079	0.001836	1.152107	0.08%
**5c-10**	-1071.327025	-1071.306577	-1071.305633	-1071.377936	0.002979	1.869351	0.05%
**5c-11**	-1071.328153	-1071.30774	-1071.306796	-1071.379079	0.001836	1.152107	0.03%
**5c-12**	-1071.327364	-1071.306777	-1071.305833	-1071.378811	0.002104	1.320280	0.02%
**5a-13**	-1071.327765	-1071.307473	-1071.306529	-1071.378811	0.002104	1.320280	0.10%
**5a-14**	-1071.33102	-1071.310739	-1071.309795	-1071.380915	0.000000	0.000000	0.02%
**5a-15**	-1071.324986	-1071.304157	-1071.303213	-1071.377435	0.003480	2.183733	0.04%

E, *E′*, *H*, *G*: total energy, total energy with zero point energy (ZPE), enthalpy, and Gibbs free energy
